# Dispersal Limitation Plays Stronger Role in the Community Assembly of Fungi Relative to Bacteria in Rhizosphere Across the Arable Area of Medicinal Plant

**DOI:** 10.3389/fmicb.2021.713523

**Published:** 2021-08-16

**Authors:** Guozhuang Zhang, Guangfei Wei, Fugang Wei, Zhongjian Chen, Mingjun He, Shuo Jiao, Yong Wang, Linlin Dong, Shilin Chen

**Affiliations:** ^1^Key Laboratory of Beijing for Identification and Safety Evaluation of Chinese Medicine, Institute of Chinese Materia Medica, China Academy of Chinese Medical Sciences, Beijing, China; ^2^Wenshan Miaoxiang Notoginseng Technology, Co., Ltd., Wenshan, China; ^3^Institute of Sanqi Research, Wenshan University, Wenshan, China; ^4^Hainan Branch Institute of Medicinal Plant, Chinese Academy of Medical Sciences and Peking Union Medical College, Wanning, China; ^5^State Key Laboratory of Crop Stress Biology for Arid Areas, College of Life Sciences, Northwest A & F University, Yangling, China

**Keywords:** rhizosphere microbiota, community assembly, environmental threshold, co-occurrence network, *Panax notoginseng*

## Abstract

Understanding the ecological patterns of rhizosphere microbial communities is critical for propelling sustainable agriculture and managing ecosystem functions by exploiting microorganisms. However, this knowledge is still unclear, especially under host-associated large-scale and regarding the comparison between bacteria and fungi. We examined community assembly processes and community characters including environmental thresholds and co-occurrence patterns across the cultivatable area of *Panax notoginseng* for bacteria and fungi. Both are vital members of the rhizosphere but differ considerably in their life history and dispersal potentiality. Edaphic factors drove the parallel variations of bacterial and fungal communities. Although bacterial and fungal communities exhibited similar biogeographic patterns, the assembly of fungi was more driven by dispersal limitation than selection compared with bacteria. This finding supported the ‘size-dispersal’ hypothesis. pH and total nitrogen respectively mediated the relative importance of deterministic and stochastic processes in shaping bacterial and fungal communities. In addition, fungal communities exhibited potentially broader environmental thresholds and more modular co-occurrence patterns than bacteria (bacteria: 0.67; fungi: 0.78). These results emphasized the importance of dispersal limitation in structuring rhizosphere microbiota and shaping community features of ecologically distinct microorganisms. This study provides insights into the improved prediction and management of the key functions of rhizosphere microbiota.

## Introduction

Plant and associated microbes are widely known as holobionts (Bordenstein and Theis, 2015); rhizosphere microbial communities play crucial roles in growth, health, and evolution of their host plant, as well as the biogeochemical cycling (Brunel et al., 2020). Bacteria and fungi, which can interact with plant negatively, positively, or neutrally, are both pivotal components of the rhizosphere biosphere (Huang et al., 2014; Tedersoo et al., 2014). Extensive studies have revealed that various factors drove the dynamics of rhizosphere microbial community, such as root exudates, edaphic factors, host genotype, and growth stage (Sasse et al., 2018; Zhang et al., 2018e). However, these studies mostly focused on the selective effects at the local scale. The macroecological patterns and community features, such as environmental responses and co-occurrence patterns of rhizosphere microbial communities at large spatial scale, especially with regard to the comparison between bacteria and fungi, are still poorly understood (Brunel et al., 2020). These patterns and features are essential to fully appreciate the formation of plant-microbe holobionts and to promote sustainable agriculture by manipulating microbes (Dini-Andreote and Raaijmakers, 2018).

From the metacommunity perspective, microbial community assembly is a comprehensive result of deterministic and stochastic processes, including selection, dispersal limitation (working in concert with drift), homogenizing dispersal, and drift (acting alone) (Leibold et al., 2004; Stegen et al., 2013; Vellend, 2010). At a large spatial scale, homogenizing dispersal and drift may play a small role in community assembly (Wu et al., 2018). Selection and dispersal limitation are therefore the main processes governing microbial community assembly across a large space. Despite that both processes play vital roles in establishing and maintaining local community, their relative importance differs in diverse habitats and organisms (Jiao et al., 2020; Stegen et al., 2012; Wu et al., 2018; Zhang et al., 2018a). Two hypotheses, namely, the “size-dispersal” and “size-plasticity” hypotheses, emphasize the importance of dispersal ability and metabolic plasticity in determining the community assembly, respectively (Farjalla et al., 2012). Bacteria tend to have higher metabolic flexibility than fungi, and the body size and propagule size of bacteria are generally smaller (Powell et al., 2015). Thus, according to the “size-dispersal” hypothesis, bacterial community assembly is expected to be more governed by selection than dispersal limitation because of their higher dispersal potentiality compared with fungi. In the “size-plasticity” hypothesis, however, the relative strength of selection in bacterial community assembly is likely weaker in comparison with that in fungal community assembly (Farjalla et al., 2012).

The response threshold of microorganisms to environment is an important indicator for predicting their abundance and distribution across complex environment gradients (van der Linde et al., 2018). Nevertheless, research on change points in environment gradients of rhizosphere microbial taxa, especially in consideration of the abundance, occurrence, and directionality of their responses, is still lacking (van der Linde et al., 2018). In view of the potentially higher environmental adaptation of bacteria, bacterial community may exhibit broader environmental thresholds compared with fungi (Jiao and Lu, 2020a). Besides being commonly affected by environmental conditions, microorganisms also link with each other universally through various types of interactions, such as mutualism, competition, commensalism, and amenalism (Faust and Raes, 2012). These interactions can impact the chemical context of the rhizosphere and thus have strong effects on root growth and health (Finkel et al., 2020). Network-based co-occurrence pattern analysis has recently been used in various habitats and has provided new insights into microbial links with one another (Lima-Mendez et al., 2015; Jiao et al., 2016; Ma et al., 2020). Similar to social and biological networks, modular structure (i.e., networks can be divided into tightly intra-linked clusters) is prevalent in microbial graphs (van der Heijden and Hartmann, 2016), thereby potentially representing the clustering of taxa with overlapping niche preference (Lima-Mendez et al., 2015). Modules may provide key ecological and biological functions as a unit (Wang et al., 2017). Revealing the modular structure and the underlying mechanisms shaping the modularity can thus produce valuable insights into the complex polymicrobial interactions and the potential function provided by certain clusters (Layeghifard et al., 2017).

Given that the ecological process and co-occurrence patterns are inherently scale-dependent (Leibold et al., 2004; Galiana et al., 2018), plant cultivatable area represents an ideal spatial scale, because it can capture complex environment gradients and spatial structures, across which rhizosphere bacterial and fungal communities are established (Soberon and Nakamura, 2009). We carried out a large-scale survey on bacterial and fungal communities in the rhizosphere of cultivated *Panax notoginseng* (Burkill) F. H. Chen across the Yunnan and Guangxi Provinces in southwestern China. The sampling area almost covers the cultivatable area of the plant. *P. notoginseng*, known as Sanqi in China, is a famous and precious perennial medicinal plant belonging to Araliaceae ginseng species with high medicinal and economic values (Jiang et al., 2020). The root of *P. notoginseng* is widely used in various prescriptions due to its major bioactive compounds, ginsenosides and notoginsenosides. Ginsenosides and notoginsenosides are steroid-like compounds with diverse pharmacological properties, such as protecting cardiovascular system, immunoregulation, anti-atherosclerotic activity, anti-tumor activity, antioxidant activity, and hemostatic activity (Wang et al., 2016). The annual output values of *P. notoginseng* has exceeded 10 billion dollars (Fan et al., 2020). Like many valuable medicinal plants, however, *P. notoginseng* is facing contradictions between increasing market requirements and limited resource caused by cultivation difficulty (Canter et al., 2005). Specifically, the arable area of *P. notoginseng* is restricted to the mountain areas in southwestern China due to its specific ecological requirements (Guo et al., 2010). Besides, the cultivation of *P. notoginseng* suffers from several replanting diseases which decrease the production quality and yields and further restrict the development of *P. notoginseng* industry (Fan et al., 2020). A systematical understanding of the ecology of rhizosphere microbiota will provide insights into the alleviation of the cultivating dilemma of medicinal plants (Soberon and Peterson, 2005). In the present study, we aimed to do the following: (i) evaluate the relative contribution of selection and dispersal limitation underlying the community assembly of bacteria and fungi and corresponding mediators; (ii) identify the environmental thresholds of bacterial and fungal assemblies; and (iii) estimate the co-occurrence patterns of bacterial and fungal communities.

## Materials and Methods

### Study Area and Sampling

Sampling was conducted in October 2017, three years after *P. notoginseng* had grown, but before harvest. All fields were managed according to the Good Agricultural Practice (GAP) (Zhang et al., 2010). In brief, virgin soil was selected to cultivate *P. notoginseng*. Before planting, 37.5 ton/ha compost was applied to soil as base fertilizer. Compound fertilizer was applied twice a year as topdressing before florescence and wintering period, respectively; the proportion of fertilizers was dependent on the growth year of *P. notoginseng*. All fields were covered with shade trellises as strong light could inhibit the growth of *P. notoginseng*. The luminousness of shade trellises was modified from 10% to 20% as plant growing. The weeds in fields were pulled out manually. Compound pesticides that contain broad-spectrum active ingredients including carbendazim and imides were sprayed mainly in April and May of each year to prevent plant diseases like root rot and blackspot.

**FIGURE 1:**
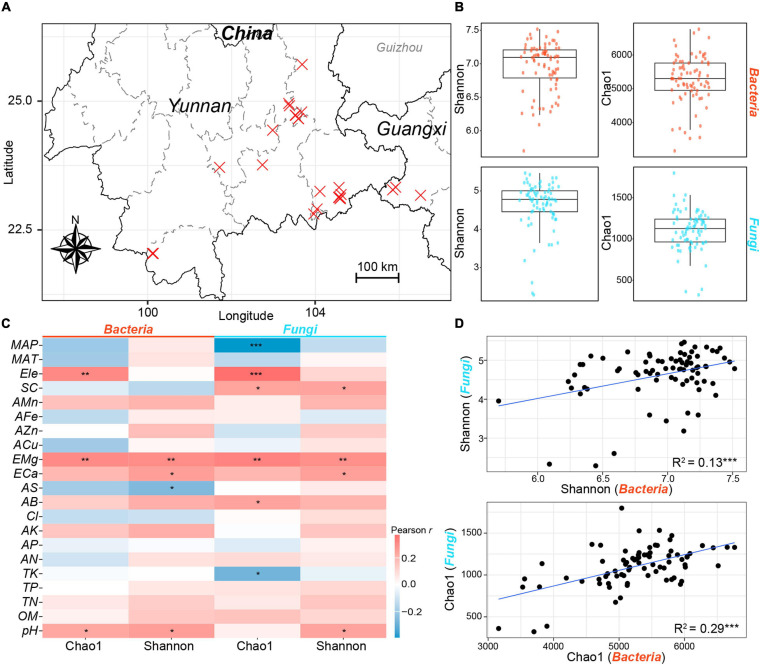
Sampling map and α-diversities of rhizosphere bacterial and fungal communities. **(A)** The location of sampling sites. Each cross represents a sampling site. The x and y axes represent longitude and latitude, respectively. **(B)** The Shannon and Chao1 indices of bacterial (red) and fungal (blue) communities. **(C)** The Pearson correlation coefficients between α-diversity indices of bacterial and fungal communities and environmental variables. Red and blue cells represent positive and negative correlations, respectively. **(D)** The linear relationships between the α-diversity indices of bacterial and fungal communities. *, *P* < 0.05; **, *P* < 0.01; ***, *P* < 0.001. The abbreviations of variables accord to the section “MATERIALS AND METHODS.”

Rhizosphere soil samples were collected from 26 sites with the median value of inter-site distance of 208 km. These sites ranged from 22.04°N to 25.71°N and 100.11°E to 106.51°E and covered the main arable area of *P. notoginseng* (Figure 1A) (GBIF., 2019; Qin et al., 2016). Ten healthy plants were collected in each of three 1.4 × 8.0 m^2^ adjacent plots at each site, and corresponding rhizosphere soil samples were combined to generate one soil sample for further physiochemical and molecular analysis. Specifically, the plant root was dug out using a sterilized shovel, and the soil loosely attached to the root was removed. Then the soil tightly attached to the root was collected using a sterile ziplock bag (Shi et al., 2019). Soil was sieved (< 2 mm) and homogenized after visible leaf and root residues being removed. In total, 78 soil samples were collected. The soil samples were transported to laboratory on ice. A subset of rhizosphere soil samples was stored at −80°C until DNA extraction while others were analyzed for edaphic factors.

Eighteen edaphic properties were measured according to previous studies (Cao et al., 2017; Shi et al., 2018; Xu et al., 2020). Soil pH was measured using a pH monitor (fresh soil water ratio 1:5) (Shi et al., 2018). Soil organic matter was determined by potassium dichromate–sulfate colorimetric method. Total nitrogen (TN) was determined by the Kjeldahl method (Xu et al., 2020). Through HF and HClO_4_ digestion, Total phosphate (TP) and total potassium (TK) were measured using molybdenum blue method and flame photometry, respectively (Shi et al., 2018). Available nitrogen (AN), available phosphorus (AP), and available potassium (AK) were subjected to the alkali diffusion method, double acid method and flame photometry respectively (Xu et al., 2020). Content of chloridion (Cl) was measured using silver nitrate titration method. Available boron (AB) and available sulfur (AS) were determined using curcumin colorimetry and barium sulfate turbidimetry, respectively (Cao et al., 2017). Exchangeable calcium (ECa), exchangeable magnesium (EMg), available copper (ACu), available zinc (AZn), available iron (AFe), and available manganese (AMn) were measured by atomic absorption spectrophotometry (Xu et al., 2020). ECa and EMg were extracted using ammonium acetate while others were extracted by DTPA. Soil conductivity (SC) was determined using conductivity monitor (dry soil water ratio 1:5) (Shi et al., 2018). Mean annual temperature (MAT) and mean annual precipitation (MAP) of all sampling sites were obtained from the Worldclim database^1^.

### Amplicon Analysis

Soil microbial communities were profiled using high-throughput sequencing of marker genes. Specifically, genomic DNA was extracted from soil samples using the FastDNA SPIN Kit for soil (MoBio Laboratories, Inc., United States). Polymerase chain reaction (PCR) assays were conducted by using the 27F/338R and ITS1F/ITS2R primer pairs for bacteria and fungi, respectively (Stegen et al., 2013; Nilsson et al., 2019a). Sequencing was performed on the Illumina MiSeq PE 250 platform (Shanghai Biozeron Co., Ltd., China). After quality filtering and the removal of chimeric sequences, the obtained sequences were clustered into operational taxonomic units (OTUs) at the 97% similarity threshold (Edgar et al., 2011; Bolyen et al., 2019). OTUs with less than 10 reads in all samples were removed to reduce potential PCR/sequencing error (Toju et al., 2016). Taxonomy was then assigned to each OTU using the RDP classifier trained on the SILVA database (release 132) and UNITE database (release 7.1) for bacteria and fungi (Pruesse et al., 2007; Wang et al., 2007; Nilsson et al., 2019b). The OTU tables were resampled to 18592 and 25645 sequences per sample for bacteria and fungi, respectively, to make the data sets (bacteria or fungi) to the same sampling effort for diversity comparisons. Aligned sequences of bacterial representative OTUs were used to construct a maximum-likelihood tree in FastTree (Tripathi et al., 2018). Given the unreliability of fungal ITS region in multiple sequence alignment, the phylogenetic tree of fungi was constructed based on the ghost-tree (Silva 132) following Fouquier et al. (2016)^2^.

### Statistical Analysis

#### α-Diversities of Bacterial and Fungal Communities

Shannon index and Chao1 index were calculated in the “vegan” package to estimate the α-diversities of bacterial and fungal communities (Jari Oksanen et al., 2019). The correlations between α-diversity indices and environmental variables were calculated using Pearson correlation analysis. Linear least squares regression was performed to evaluate the relationships between α-diversity indices of bacteria and fungi.

#### Community Variations and Environmental Thresholds

Variation partition analyses (VPA) were performed to quantify the effects on composition variations of bacterial and fungal communities of three partitions, namely, edaphic chemistry, climatic variables, and spatial factors (van der Linde et al., 2018). Community variation was represented by pair-wise Bray-Curtis dissimilarities. Elevation, MAT, and MAP were grouped into climatic factors, whereas spatial variables were extracted based on principal coordinates analysis of the neighbor matrices (PCNMs), which explicitly allowed the introduction of a spatial relationship into statistical models; PCNMs were obtained using the pcnm function in the “vegan” package (Dray et al., 2006). Variables in each partition were selected through forward-selection procedures with distance-based redundancy analysis based on the criteria of adjusted *R*^2^ and *P* < 0.05 using ordiR2step function in “vegan” package (Jari Oksanen et al., 2019).

Based on the results of VPA, edaphic chemistry was the most important environmental driving forces for the variations of bacterial and fungal communities. To explore the main edaphic factors determining community composition, we performed non-metric multi-dimensional (NMDS) using metaMDS function in the “vegan” package. Environmental factors were then fitted to the ordination with envfit function in the “vegan” package (van der Linde et al., 2018). Factors with *R*^2^ > 0.2 and *P* < 0.01 were selected as key variables for each model. Variable clustering was used to assess collinearity, and variables with absolute spearman ρ > 0.7 were discarded (Supplementary Figure 2). We further detected the community thresholds of bacterial and fungal communities for key variables using threshold indicator taxa analysis in the “TITAN2” package (Baker and King, 2010). Specifically, the sum of standardized indicator value scores (z-scores) of each OTU was obtained to generate the lower and upper community-level threshold for each variable.

Multivariate regression and variance decomposition were conducted to explore important predictors of abundance patterns for dominant taxonomic groups and fungal functional guilds. Fungal functional guilds were annotated using the python script FUNGuild.py (Nguyen et al., 2016). Regression models were selected based on adjusted *R*^2^ and Schwartz’s information criterion (BIC) using regsubsets function in the “leaps” package (Lumley, 2020).

#### The Estimation of Community Assembly Processes

Distance-decay relationships (DDRs) were quantified as the slopes of ordinary least-squares regressions between community similarities (1 – Bray-Curtis dissimilarities) and geographic distance. To clarify the relative importance of selection and dispersal limitation in bacterial and fungal communities, we used the above-described variation partitioning and null model analysis. These methods were complementary to one another (Zhou and Ning, 2017). For variation partition analysis, the strength of selection was represented by pure environmental variation without a spatial component, i.e., the effects of soil and climate partitions after excluding space partition. Pure spatial variation without other components represented the effect of dispersal limitation. Then, the ratio of selection to dispersal limitation effect (SDER) was calculated for cross-system comparison (Wu et al., 2018).

Null model analysis was performed based on the framework of Stegen to divide community pairs into the underlying driving forces of selection (variable or homogeneous), dispersal limitation, homogenizing dispersal, and undominated (Stegen et al., 2015). This framework integrated phylogenetic and taxonomic variations using null model-based β-diversity metrics, i.e., β-nearest taxon index (βNTI) and Raup-Crick based on Bray-Curtis (RC_Bray_). Significant deviation of phylogenetic diversity from null expectation (| βNTI| > 2) represented the effect of selection, in which βNTI < −2 indicates homogeneous selection, whereas βNTI > 2 indicated variable selection. RC_Bray_ was further used to classify the remaining community pairs with | βNTI| < 2. Specifically, the effect size of dispersal limitation was quantified as the fractions of community pairs with RC_Bray_ > 0.95, whereas the percentage of RC_Bray_ < −0.95 was identified as the influence of homogenizing dispersal. As the phylogenetic turnover in null model analysis was quantified among closest relatives, we tested phylogenetic signals for both communities before analysis, in accordance with a previous description (Tripathi et al., 2018; Supplementary Figure 3). To make the above two frameworks comparable, we assessed the SDER using the fractions of pairwise communities dominated by selection divided by the percentage of community pairs governed by dispersal limitation.

To verify the robustness of fungal phylogeny described above in comparing the SDER between bacteria and fungi, we further constructed a phylogeny for fungi using the perl script taxonomy_to_tree.pl (Tedersoo et al., 2018) and calculated the community assembly processes and SDER using Stegen’s null model based on this new tree. We also estimate the contribution of stochasticity to the assembly of bacterial and fungal communities based on only taxonomic null model (i.e., RC_Bray_) (Chase et al., 2011; Stegen et al., 2013; Gao et al., 2020).

Sloan’s neutral model was used to estimate the potential contribution of neutral processes to community assembly by fitting the frequency with which microbial taxa occur in a set of local communities and their abundance in the metacommunity (Burns et al., 2016). The single free parameter in this neutral model was the migration rate *m*, which represented the probability that a random loss of an individual in a local community would be replaced by dispersal from the metacommunity, and therefore, can be used to evaluate dispersal limitation. Lower *m*-values indicated that microbial assemblies are more limited by dispersal (Burns et al., 2016). The overall fit of the neutral model was calculated by comparing the sum of squares of residuals, *SS*_eer_, with the total sum of squares, *SS*_total_: generalized *R*^2^ = 1 – *SS*_eer_/*SS*_total_. To estimate whether the performance of neutral model was better than random sampling from source metacommunity, we compared the neutral with binomial models based on Akaike information criterion. Sampling from a binomial distribution represented that local communities are random subsets of the metacommunity in the absence of dispersal limitations and drift (Jiao et al., 2020). The *R* code here was used according to Burns et al. (2016).

We also estimated the niche breadth and dispersal ability, which were traits of great concern, because they influence the relative importance of selection versus dispersal limitation. Niche breadth represented potential metabolic flexibility and was computed using Levin’s niche breadth index (*B*) (Pandit et al., 2009). Dispersal ability at community level (*D*) was calculated in a relative sense using the average of pairwise shared proportion of sequence numbers of each OTU (Wu et al., 2018).

Finally, to explore the main factors mediating the assembly process of bacterial and fungal communities, we tested the variation in community assembly process along the environmental gradients using Stegen’s framework (Stegen et al., 2015). We used the Mantel test to compare all pairwise comparisons of βNTI values with the Euclidean distance matrices of each variable. A partial Mantel test was then performed to estimate the relationship between phylogenetic variations and main factors after controlling for geographical distance and other variables. The correlation was visualized using Mantel correlograms. These procedures were performed by means of mantel and mgram functions in the “ecodist” package (Goslee and Urban, 2007).

#### Co-occurrence Patterns Inference

Before network inference, rare OTUs with relative abundance of < 0.01% were removed to mitigate zero inflation. We used a conservative method to construct co-occurrence networks with seeing bacteria and fungi as a whole (Ma et al., 2020). Spearman correlation and Bray-Curtis dissimilarity measures were selected to infer interactions among taxa. The *P*-values for each measure were computed as the probability of the null value obtained from permutation under a Gaussian curve, which is generated from the mean and standard deviation of bootstrap distributions. For Spearman correlation, a renormalization step was conducted after each permutation to ease the compositionality problem. The *P*-values of the two measures were then combined based on Brown’s methods (Poole et al., 2016) and corrected using BH method (Benjamini and Hochberg, 1995). After filtering out links with the adjusted *P*-value of > 0.05, the Spearman correlation coefficient threshold (0.74) was determined based on the random matrix theory (Deng et al., 2012). Bacterial and fungal metacommunity networks were extracted from the whole network using subgraph function in “igraph” package (Csardi and Nepusz, 2006). Modules in networks were detected using fast greedy clustering method (Clauset et al., 2004), and modularity was calculated using the modularity function in the “igraph” package. The first principal component (PC1) of dominant modules (also known as eigengene) was calculated to determine the potential niche preference. Networks were visualized using the Gephi platform (Bastian et al., 2009).

Then, we generated local networks for each plot according to the nodes retained in corresponding samples. Network level topological features, including graph density, clustering coefficient, centrality measures, modularity, graph diameter, average shortest length, and number of nodes and edges, were calculated in “igraph” packages. After removing features with strong collinearity, we generated pairwise Euclidean distance among samples using standardized values. The spatial variation rate of co-occurrence patterns was quantified using the ordinary least-square regression of Euclidean distance of network to geographic distances. Multiple regression on distance matrices (MRMs) were then used to determine key factors potentially affecting co-occurrence patterns using MRM function in “ecodist” package (Goslee and Urban, 2007). All standardized Euclidean distances of variables without strong collinearity were introduced into the first MRM model. Then, significant (*P* < 0.05) predictors were retained in the second model to obtain results.

All statistical analyses were conducted in R (v3.5.3) (Team, 2019), using “vegan” (Jari Oksanen et al., 2019), “TITAN2” (Baker and King, 2010), “leaps” (Lumley, 2020), “relaimpo” (Grömping, 2006), “MASS” (Venables and Ripley, 2013), “Hmisc” (FE, 2020), “igraph” (Csardi and Nepusz, 2006), “WGCNA” (Langfelder and Horvath, 2008), “ggplot2” (Wickham, 2016), “picante” (Kembel et al., 2010), “minpack.lm” (Elzhov et al., 2016), and “ecodist” (Goslee and Urban, 2007) packages. In analyses sensitive to data normality, variables except pH were logarithmically transformed as needed.

### Data Availability

The raw sequencing data are publicly available in the NCBI Sequence Read Archive (SRA) under the Bioproject number PRJNA559079. R codes on the statistical analyses are available at https://github.com/githubzgz/Panax.notoginseng.

## Results

### Diversities of Rhizosphere Bacterial and Fungal Communities

After filtering out OTUs with less than 10 reads in all samples, a total of 4,931,855 and 4,658,981 reads were retained for bacterial and fungal communities, respectively. The number of bacterial sequences per sample ranged from 18,592 to 154,413, while the number of fungal sequences in each sample was from 25,645 to 71,702. After subsampling to the minimum number of reads per sample (bacteria: 18592; fungi: 25645), we obtained 14,447 and 5,988 bacterial and fungal OTUs, respectively. Although individual samples of bacteria and fungi did not show a full rarefaction saturation (Supplementary Figure 1A), the rarefaction curve of the pooled data (78 samples) of two domains were saturated, suggesting that we had good coverage of the bacterial and fungal richness at global level (Supplementary Figure 1B).

The α-diversities of bacterial communities were far higher than those of fungal communities as indicated by the Shannon index and Chao1 index (Figure 1B). Pearson correlation analysis showed that the two α-diversity indices of bacterial communities were significantly correlated with pH (Shannon, *r* = 0.26, *P* < 0.05; Chao1, *r* = 0.23, *P* < 0.05) and EMg (Shannon, *r* = 0.30, *P* < 0.01; Chao1, *r* = 0.30, *P* < 0.01); while the α-diversity indices of fungal communities exhibited significant correlations with EMg (Shannon, *r* = 0.29, *P* < 0.01; Chao1, *r* = 0.33, *P* < 0.01) and SC (Shannon, *r* = 0.24, *P* < 0.05; Chao1, *r* = 0.24, *P* < 0.05) (Figure 1C). Besides, the linear least-square regression indicated that significant and positive linear relationships exhibited between the α-diversity indices of bacterial and fungal communities (Shannon, *R*^2^ = 0.13, *P* < 0.001; Chao1, *R*^2^ = 0.29, *P* < 0.001). These results suggested that similar environmental variables might drive the covariation of the α-diversities of bacterial and fungal communities.

### The Key Driving Forces of Community Variations and Environmental Thresholds

**FIGURE 2:**
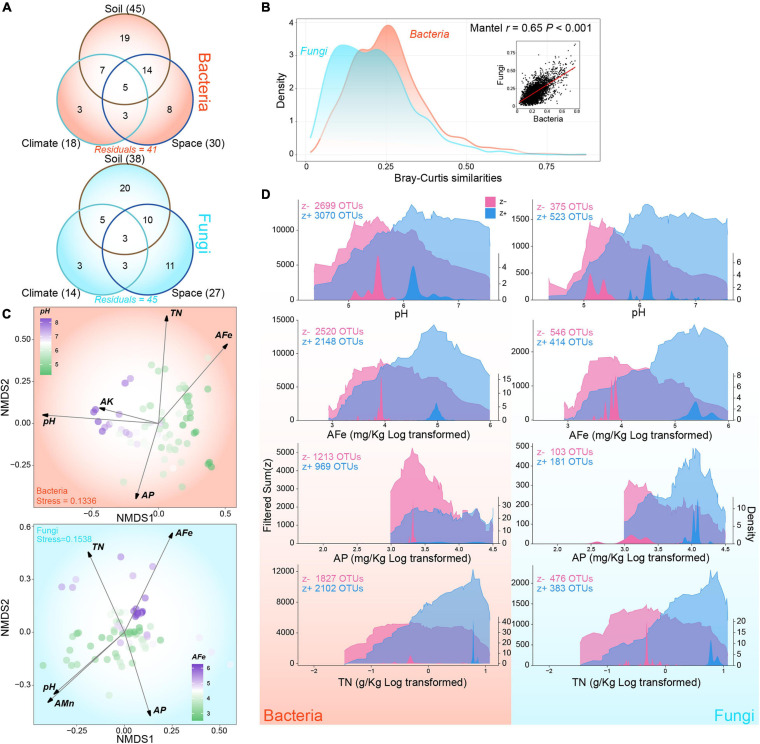
Composition variations and environmental thresholds of rhizosphere bacterial and fungal communities. **(A)** Variation-partitioning Venn diagram showing the proportions of individual and combined contributions of edaphic, climatic, and spatial factors. Values in brackets indicate the total proportion of variance explained by corresponding partition. **(B)** The relationship between pair-wise similarities of bacterial and fungal communities based on Bray-Curtis index. Area graphs show the density distribution of bacterial and fungal community similarities. Dot plots represents the correlation between bacterial and fungal community similarities. **(C)** Global non-metric multidimensional scaling ordination of community composition and fitted key variables of bacteria and fungi. **(D)** Cumulated community thresholds for key variables of bacteria and fungi. Left axes and the transparent area graph indicate the sum of filtered z– and z + at each change points while right axes and non-transparent area graph represent the density of change points. Variables are log transformed for better visualization as needed. The abbreviations of variables accord to the section “MATERIALS AND METHODS.”

Forward-selected models based on Bray-Curtis dissimilarities explained 59% and 55% of the variance in β-diversities of bacterial and fungal communities, respectively (Figure 2A and Supplementary Table 1). In the three parts, soil explained maximum variance in the bacterial and fungal models (bacteria: 45%; fungi: 38%), followed by space (bacteria: 30%; fungi: 27%), and climate (bacteria: 18%; fungi: 14%). In addition, significant co-variation existed in the pair-wise similarities of bacterial and fungal communities (Mantel *r* = 0.65, *P* < 0.001), although the between-sample similarities in fungal communities were much lower than those in bacterial communities (Figure 2B). Global NMDS were performed to visualize the variations in community composition (Figure 2C). After fitting environmental variables to the ordinations, we identified six key edaphic factors, as follows: pH, AFe, AP, and TN for both bacteria and fungi; AK for bacteria only; and AMn for fungi only (Figure 2C and Supplementary Table 2). Soil pH and AFe were the most influential variables for bacteria and fungi separately. The influences of environmental factors on fungi were more uniform than those on bacterial communities (Figure 2C and Supplementary Table 2).

For the four key edaphic factors shared by bacterial and fungal communities, environmental thresholds were identified through cumulating decreasing (z−) and increasing (z +) change points, which were pure and reliable (Figure 2D). For pH, we detected that the sum(z−) peaked at 5.53 for bacteria and 5.33 for fungi; the sum(z−) peaked at 6.18 for both. For AFe, we found peaks at 50.48 and 143.19 mg kg^–1^ for sum(z−) and sum(z +) in bacterial communities, whereas we found peaks at 48.09 and 222.01 mg kg^–1^ for sum(z−) and sum(z +) in fungal communities, respectively. For AP, we detected the community-level decreasing threshold at 27.73 mg kg^–1^ and the increasing threshold at 29.93 mg kg^–1^ for bacteria. The sum(z−) and sum(z +) peaks were detected at 22.33 and 55.79 mg kg^–1^ for fungi. Indicator taxa showed a clear threshold of change for TN in fungal communities, with a 0.73 g kg^–1^ z− peak and a distinct 2.19 g kg^–1^ z + peak. In bacterial communities, the sum(z +) showed an obvious peak at 2.19 g kg^–1^, and the sum(z−) had a weaker peak at 0.75 g kg^–1^. The density distribution around the change points of fungal community were more dispersed relative to those of the bacterial community (Figure 2D). At the phylum level of bacteria, the abundance of OTU which responded to key factors, especially pH, TN, and AFe, was evenly distributed except for Cyanobacteria, which was dominated by taxa that showed negative response to TN and AFe (Supplementary Figure 4A). For fungal phylum, OTUs which responded positively to pH and AFe and negatively to TN accounted for the largest proportion in Ascomycota, Basidiomycota, and Mucoromycota, respectively (Supplementary Figure 4B). Plant pathogen showed different response patterns compared with saprotroph and symbiotroph. The plant pathogen was dominated by OTUs’ positive response to pH, whereas the latter two were both dominated by OTUs’ negative response to TN (Supplementary Figure 4C). These results showed that the key edaphic factors driving bacterial and fungal community variations are similar, and fungal community exhibited broader thresholds for these factors than the bacterial community.

Multiple regression and variance decomposition analysis were used to identify the main predictors for dominant bacterial and fungal phyla and fungal functional guilds to obtain a more comprehensive understanding of microbial distribution (Supplementary Figure 5). AFe, MAT, MAP, AP, and pH were the variables most frequently identified to predict abundance patterns of bacterial and fungal phyla. For example, AFe was the best predictor for the distribution of Acidobacteria, Chloroflexi, Cyanobacteria, Verrucomicrobia, and Ascomycota. Meanwhile, pH and MAT were the most important variables related to the relative abundance of Rokubacteria and Gemmatimonadetes, respectively. Given the abundance distribution of different fungi guilds, the distributions of plant pathogen and saprotroph were best predicted by pH (Supplementary Figure 5).

### Relative Importance of Ecological Processes in Shaping Microbial Communities

**FIGURE 3:**
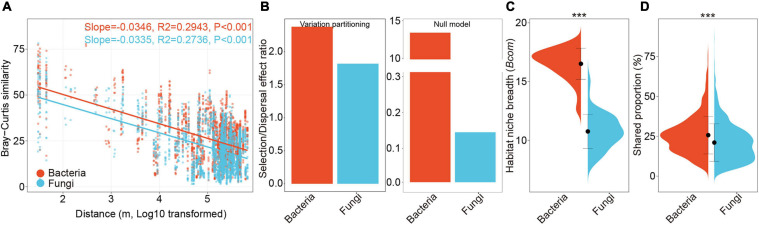
Biogeographic patterns and ecological processes of bacterial and fungal communities. **(A)** Distance-decay relationships showing Bray-Curtis similarities of bacterial and fungal communities against spatial distance among sampling sites. Solid lines denote the fitness of ordinary least-squares regressions. **(B)** Selection/dispersal limitation ratio of bacterial and fungal communities estimated by variation partitioning analyses and null model analyses. **(C)** Violin plots illustrating mean habitat niche breadth from all OTUs in each sample (*Bcom*) of bacterial versus fungal communities. Black dot represents the mean value of *Bcom* and error bars indicate standard deviation. **(D)** Violin plots showing mean shared proportions (%) of sequences of bacterial and fungal communities, which represents the potential dispersal abilities **(D)**. The significance of difference between bacteria and fungi were determined by Wilcoxon rank sum test. ***, *P* < 0.001.

We evaluated the DDRs for bacterial and fungal communities in the rhizosphere across the cultivatable area of *P. notoginseng*, which spanned a maximum geographic distance of more than 650 km (Figure 1A). Significant DDRs were found for bacteria (*R*^2^ = 0.29, *P* < 0.001) and fungi (*R*^2^ = 0.27, *P* < 0.001) (Figure 3A). The slopes of bacterial (−0.0346) and fungal (−0.0335) communities were similar, indicating a semblable decay relationship of bacterial and fungal community similarities (Figure 3A).

Variation partition analysis based on Bray-Curtis dissimilarities (Figure 2A) and Stegen’s null model analyses both revealed that the SDER of bacterial communities (VPA: 3.22, Null: 13.42) was higher than that of fungal communities (VPA: 2.25, Null: 0.15), suggesting that the relative importance of selection was higher in shaping bacterial communities (Figure 3B). The negligible proportions of homogenizing dispersal estimated by Stegen’s null model analysis confirmed its minor role in microbial community assembly at a large-scale space (Supplementary Figures 6A,B). Similar results were obtained when fungal phylogeny was constructed using taxonomy_to_tree.pl (Supplementary Figures 6C,D). The RC_Bray_-based estimation also showed that the influences of stochastic process on bacterial community assembly (5.89%) was lower than that on fungal community assembly (12.79%) (Supplementary Figure 7). The neutral models of bacterial and fungal communities both outperformed a binomial distribution model, thereby suggesting the importance of dispersal limitations (Table 1). The estimated migration rate of bacterial communities was much higher than that of fungal communities, which also supported the results of SDER (Table 1). In addition, bacterial communities exhibited significantly broader community niche breadths (*Bcom*) (*P* < 0.001, Figure 3C) and stronger dispersal abilities (*D*) (*P* < 0.001, Figure 3D) in comparison with fungal communities. These results indicated that fungal community assembly was more driven by dispersal limitation relative to selection compared with bacterial community assembly.

**TABLE 1:** Fit of the neutral model and comparison with binomial model.

Group	Migration rate *m*	Generalized *R*^2^	Akaike information criterion	
			
			Neutral model	Binomial model
Bacteria	0.246	0.562	−17824.530	−13262.250
Fungi	0.014	0.255	−5571.411	521.557

**FIGURE 4:**
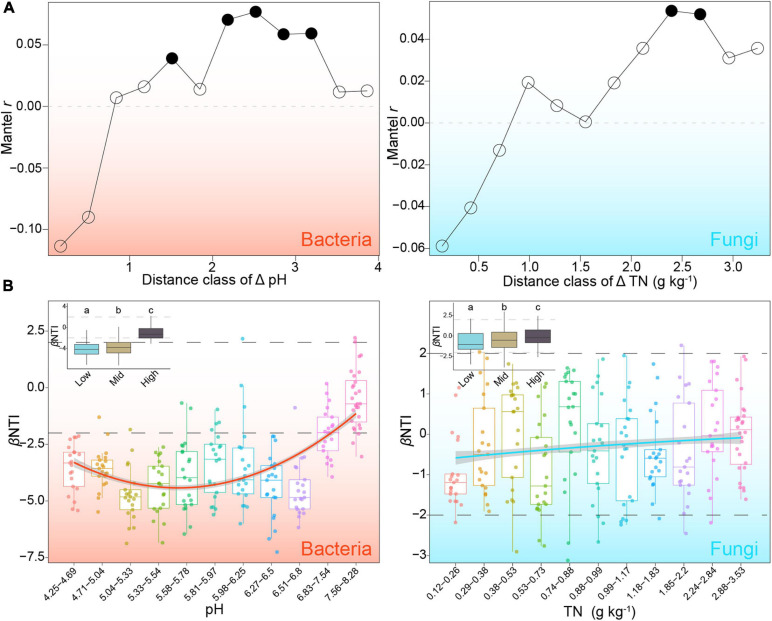
Main factors mediating the relative contributions of stochastic and deterministic processes to bacterial and fungal community assembly. **(A)** Mantel correlograms of β-nearest taxon index (βNTI) against distance classes of key variables (i.e., pH for bacteria and TN for fungi) identified by mantel and partial mantel tests. Solid dots represent significant correlation at corresponding distance class. **(B)** Patterns of βNTI across different sub-groups along gradients of pH for bacteria and TN for fungi. Solid lines represent least-square regression with a second order term.

The correlation between βNTI and environmental variables was then tested to assess the changes in the relative contributions of stochastic and deterministic processes. The results of the Mantel test showed that MAP, pH, OM, TN, AN, AK, AFe, and Cl were significantly related to bacterial phylogeny variation (*P* < 0.05), whereas TN, ACu, and AMn were remarkable predictors of fungal βNTI (Supplementary Table 3). After controlling for geographic distance and other measured environmental variables, pH was determined to be the best predictor with the highest correlation with bacterial βNTI (*r* = −0.23, *P* < 0.001) and TN remaining significant in the fungal test (*r* = −0.18, *P* < 0.001) (Supplementary Table 4). Mantel correlograms showed that βNTI of bacteria and fungi were positively related to the Euclidean distance of pH and TN at distant distance class, respectively (Figure 4A). Samples were divided into sub-groups based on the above two variables and correlated with βNTI. With increasing pH from acid to mildly alkaline, the relative importance of the stochastic process in bacterial assembly first decreased slightly and then increased obviously (Figure 4B). Along with the increasing gradients of TN, the relative influence of deterministic fungal assembly decreased slightly (Figure 4B). These results showed that pH and TN are the main factors mediating the balance in ecological processes underlying bacterial and fungal community assembly, respectively.

### Co-occurrence Pattern of Bacterial and Fungal Communities at Regional and Local Levels

**FIGURE 5:**
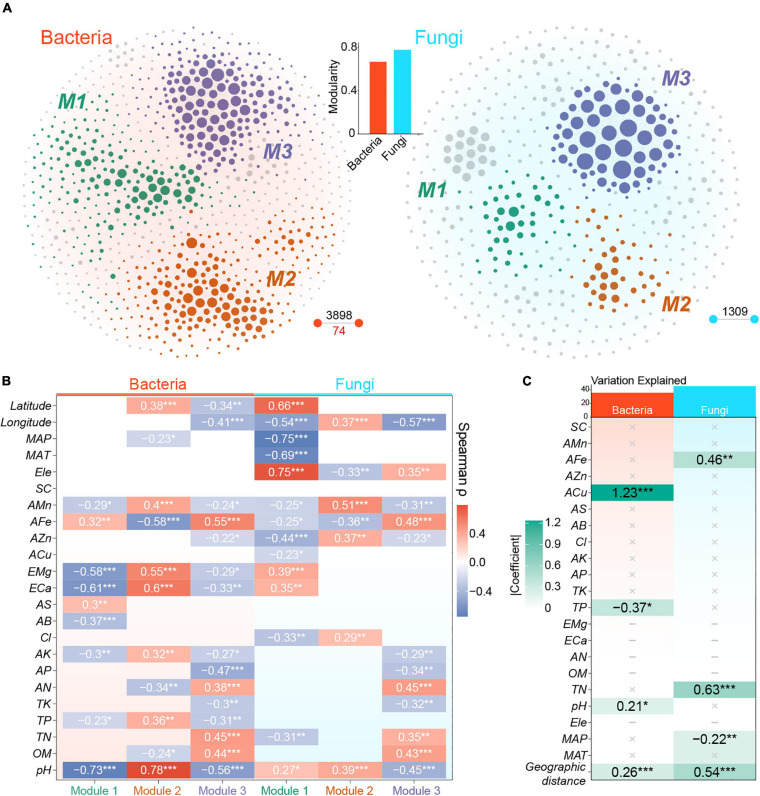
Co-occurrence patterns of bacterial and fungal communities. **(A)** The co-occurrence networks of bacteria and fungi extracted from cross-domain metacommunity network. Nodes and lines represent OTUs and robust links among OTUs, respectively. Nodes are colored according to dominant modules. Black digits represent the number of positive links, whereas red digit represents the number of negative links. The bar plot represents the modularity of bacterial and fungal metacommunity networks. **(B)** Spearman correlations between PC1 of modules and measured variables. Modules of bacterial and fungal metacommunity networks are defined in Figure 4A. (**C**) The final result of multiple regression on the Euclidean distance of graph-level topological features of local networks. ‘×’ represents non-significant variables and ‘–’ represents variables with strong collinearity. Tile colors are mapped to the absolute values of standardized coefficients. *, *P* < 0.05; **, *P* < 0.01; ***, *P* < 0.001.

The cross-domain and bacterial and fungal metacommunity co-occurrence networks captured 5798, 3972, and 1309 links among 1421, 905, and 492 nodes, respectively. All three networks followed roughly scale-free degree distribution (Supplementary Figures 8A,B). Positive covariations dominated rhizosphere metacommunity networks, and all negative links were identified among bacterial nodes (Figure 5A). Node-level topological features showed that bacterial nodes were significantly more connected than fungal nodes in the cross-domain meta-community network (Supplementary Figure 8C). In the six main modules identified by fast greedy modularity optimization algorithm, three modules (1, 2, and 5) were dominated by bacterial OTUs (> 85%), whereas module 6 was almost entirely composed of fungal nodes (94%, Supplementary Figure 9A). Interactions between bacteria and fungi predominantly occurred in modules 3 and 4, and these were contributed by bacterial phyla, such as Chloroflexi, Proteobacteria, and Acidobacteria, and fungal class, such as Sordariomycetes, Mortierellomycetes, and Eurotiomycetes (Supplementary Figure 9B).

Fungal metacommunity network was more modular than bacterial metacommunity network (bacteria: 0.67; fungi: 0.78; Figure 5A). Three dominant modules with number of nodes of more than 10% existed in both networks (Figure 5A). The correlation between module eigengene and environmental variables revealed different environmental preferences of modules. Bacterial module 1 showed a strongly negative correlation with soil pH (ρ = −0.73), whereas module 2 showed the contrary (ρ = 0.78). In addition, module 3 of bacterial metacommunity network showed significant but weaker relevance with AFe (ρ = 0.55), pH (ρ = −0.56), and TN (ρ = 0.45). There was a strong correlation between fungal module 1 and MAP (ρ = −0.75), which was highly correlated with the latitude (ρ = −0.85) (Supplementary Figure 2). Other fungal modules, however, exhibited weaker correlations with environmental variables, and the most powerful relevance was shown between module 2 and AMn (ρ = 0.51) and between module 3 and AFe (ρ = 0.48, Figure 5B).

A set of local networks were generated through the remaining nodes in each sample. After removing features with strong collinearity, 11 network-level topological features were maintained as representations of the co-occurrence patterns of both bacteria and fungi (Supplementary Figure 10). The final MRM models showed that ACu, TP, geographic distance, and pH were significant variables that explained the variation in bacterial co-occurrence pattern, whereas TN, geographic distance, AFe, and MAP were remarkable variables in the fungal model (Figure 5C). The MRM model of fungi explained more variations (*R*^2^ = 0.45) in co-occurrence pattern than that of bacteria (*R*^2^ = 0.38, Figure 5C). For key variables selected by MRM model, ACu was significantly and positively correlated with degree centrality, graph density, and clustering coefficient of bacterial local networks, but it had negative correlations with average shortest length, graph diameter, eigenvector centrality, and modularity (Supplementary Figure 11). TN was positively associated with a series of fungal topological properties, such as graph density, closeness centrality, and number of edges. Furthermore, eigenvector centrality and modularity of fungal networks showed significant and negative correlation with TN (Supplementary Figure 11). In addition, the ordinary least-square regression of Euclidean distance of fungal network features to geographic distance showed a deeper slope (−0.10) than those of bacteria (−0.03) (Supplementary Figure 12).

## Discussion

In the rhizosphere of *P. notoginseng*, edaphic factors are the most important partitions for explaining variations in bacterial and fungal communities (Figure 2A), which are consistent with the results recently reported in large-scale surveys on bacterial communities (Powell et al., 2015; Zhang et al., 2018a). However, previous studies showed that climatic factors are the best predictors of fungal community composition across a large space (Tedersoo et al., 2014; Zhang et al., 2020), possibly because the climatic gradients in our study area are much narrower than those in areas of continental or global scale. Another potential reason is that plant root imposes unique selective effects on fungal communities by changing the rhizosphere soil environment in comparison with the results of other studies (Hannula et al., 2017). The significant linear relationships in α-diversity indices and strong co-variation in pair-wise similarities of bacterial and fungal communities (Figures 1D, 2B) and the similar key influential factors (Figure 2C) provided potential evidence for the inference of rhizosphere effect (Wang X. et al., 2020); and these results were not observed in studies of natural soils (Powell et al., 2015; Liu et al., 2020).

Understanding the ecological processes structuring communities is essential for the management of microbiota (Meyer et al., 2018; Brunel et al., 2020). The neutral model emphasized the non-negligible role of dispersal limitation in the assembly of both bacterial and fungal communities (Table 1), which indicated that stochastic processes should also be considered even in a strongly selective environment such as the rhizosphere (Wang X. et al., 2020). This result was also supported by the observations in soybean rhizosphere at continental scale (Zhang et al., 2018a, d). The wider niche width of bacteria than fungi reported in our study (Figure 3C), indicated that bacteria are more likely to be generalists with potentially higher phenotypic plasticity than fungi (Langenheder et al., 2005). The lower dispersal potentiality of fungi in comparison with bacteria was also within expectations (Figure 3D), as the smaller body and propagule sizes of bacteria might allow easier passive transport compared with fungi (Farjalla et al., 2012; Powell et al., 2015). Although bacterial and fungal communities exhibited similar distance-decay relationship (Figure 3A), the variance partitioning, null model, and neutral model jointly suggested that fungal community assembly was more driven by dispersal limitation than selection compared with bacteria (Table 1 and Figure 3B, Supplementary Figure 6). This pattern implied that the influence of dispersal ability overwhelmed the effect of phenotypic plasticity on the determination of the relative importance of selection versus dispersal limitation in shaping bacterial and fungal communities (Wu et al., 2018). Our observations in the context of rhizosphere are consistent with the results of recent large-scale studies conducted in natural soils from different habitats (Powell et al., 2015; Chen et al., 2020; Wang P. et al., 2020) and provide new evidence for the ‘size-dispersal’ hypothesis that organisms with larger body size (fungi) are more dispersal limited than those with smaller body size (bacteria) (Farjalla et al., 2012). The stronger dispersal limitation in fungal community assembly also provided a potential explanation for the higher β-diversities in fungal community compared with bacterial community (Figure 2B), because dispersal limitation will increase spatial heterogeneity in species composition (Galiana et al., 2018). Considering the findings of previous studies on the comparison of ecologically distinct organisms in diverse environments like ocean and tank of bromeliads (Farjalla et al., 2012; Wu et al., 2018), this result further supported the hypothesis that the relative contributions of deterministic and stochastic assembly process are highly dependent on the ecological context.

Revealing which community assembly processes are more important under different contexts could provide us with insights into the generation and maintenance of species diversity and the formation of community structure (Jiao and Lu, 2020b). Our study showed that soil pH and TN mediated the balance between deterministic and stochastic assembly of bacteria and fungi based on phylogenic null model, respectively (Figure 4). The stochastic assembly of bacterial community plays larger role in neutral and alkalescent soils than that of fungal community (Figure 4B), and this result is supported by a previous meta-analysis conducted at global scale (Tripathi et al., 2018). A continental-scale study also showed that the assembly of abundant bacteria in agriculture soils was mediated by pH level (Jiao and Lu, 2020b). Although only weak pattern was observed, the importance of stochastic process in fungal assembly increased with the increasing concentration of soil total nitrogen (TN). This finding was not surprising, because total nitrogen is known to affect fungal community composition and diversity at multiple environments and scales (Zhang et al., 2018c; Chen et al., 2020; Shi et al., 2020). Rhizosphere soils with low concentration of TN may select fungal taxa with stronger and more diversified nutrient acquisition capacity. These results emphasized the role of certain edaphic factors in rhizosphere microbial assembly.

One unanticipated finding in our study was that fungi showed potentially broader environmental thresholds for key variables at the community level than bacteria even if bacteria tended to be more metabolically flexible (Figures 2D, Figure 2C, 3C). The community-level environmental thresholds have been considered as a measure of niche breadth associated with specialized environmental variables (Jiao and Lu, 2020a; Zhang et al., 2020). An explanation for this potential contradiction might be as follows: the change of the occurrence and abundance of taxa along environmental gradients in real world reflected the interactions between environment filtering and spatial factors rather than the sole environmental responses (Boulangeat et al., 2012). In other words, the relative lower dispersal ability among local sites amplifies the role of stochastic demographic changes in shaping abundance distribution of fungal taxa (Vellend, 2010; Dini-Andreote et al., 2015). On the other hand, low migration rate *per se* may also lead to the rarity of one taxon even in its preferred niche (Ai et al., 2013). Consequently, the relatively strong dispersal limitation can decouple the relationship between abundance distribution and environment to some extent and thus broaden the stable range of fungal community across complex environmental gradients. In contrast, the relatively high dispersal of bacteria will enhance the effects of environmental filtering in shaping species distribution landscape compared with fungi (Vellend, 2010). The more unconcentrated density distribution observed around the change points in fungal community provided evidence for this explanation (Figure 2D). This observation was also supported by a previous study in natural soils, which showed that fungi governed by a highly stochastic assembly process was largely independent of disturbance introduced by land use compared with bacteria (Powell et al., 2015).

Co-occurrence patterns are prevalent and play critical roles in understanding microbial community structure (Ma et al., 2016). In our study, positive covariations dominate the metacommunity co-occurrence network (Supplementary Figures 9A, Figure 5A), which is consistent with the findings of previous comparative studies (Shi et al., 2016; Ma et al., 2020), suggesting that extensive mutualistic interactions potentially occur among rhizosphere microbes. The universal positive co-occurrence may be associated with the abundant available nutrient secreted by plant root (Mendes et al., 2011). In the cross-domain metacommunity network, most main modules were dominated by either bacterial or fungal nodes (Supplementary Figure 9A), implying the different niche preference between most bacterial and fungal taxa (Layeghifard et al., 2017). Fungi can utilize more recalcitrant organic substrates that cannot be decomposed by bacteria (Boer et al., 2005). A study in the rhizosphere of legumes also supported our result (Zhang et al., 2018b). However, the limited number of bacteria–fungi links might represent the overlapping niche among certain taxonomic groups (Supplementary Figure 9B), which probably originated from either the similar preference for simple plant-derived compounds of bacteria and fungi or the fungal-derived bacterial niches (Boer et al., 2005).

In the comparison of single-domain meta-community networks, a more modular structure of fungal graph (Figure 5A) indicated that fungi occupied more decentralized niches than bacteria (Shi et al., 2016; Layeghifard et al., 2017). This difference might also be due to the higher spatial heterogeneity among fungal local communities caused by stronger dispersal limitation (Galiana et al., 2018), because a low-level exchange of organisms hinders the establishment of local taxa in distant fundamental niches (Stegen et al., 2013). Thus, the co-variation among fungal taxa captured by network inference might be restricted to spatially more adjacent sites with specific environmental gradients in comparison with bacteria (Faust and Raes, 2012). The weaker correlations between fungal module eigengenes and environmental variables detailed in our study (Figure 5B) further implied that the formation of fungal modules was less dependent on environment compared with bacteria (de Menezes et al., 2015). As discussed above, the absence of complete responses of fungal taxa to the whole environmental gradients within the region would weaken the correlations between fungal module eigengenes and environmental factors. In a study on sedimentary microbiota, researchers found that the more disperse-limited fungi exhibited higher modularity than bacteria in most areas (Chen et al., 2020). A drought-stress study in grassland mesocosms also provided evidence that bacterial networks were more sensitive to environment disturbance than fungal networks (de Vries et al., 2018). The fungi-dominated modules showed weaker associations with edaphic and climatic factors than bacteria-dominated modules in the soils of soybean fields (Zhang et al., 2018b). These results emphasized the potential role of neutral processes in structuring co-occurrence patterns of different organisms.

The topological patterns of local co-occurrence networks allow a more comprehensive understanding of the responses of microbial communities to local and regional factors (Ma et al., 2016). The present study showed that ACu and TN were the most important explanatory variables for the variations in local co-occurrence patterns of bacteria and fungi, respectively (Figure 4C). Copper ions are essential cofactors of various enzymes, and copper homeostasis is critical for maintaining core metabolic processes in bacteria (Rademacher and Masepohl, 2012; Kenney et al., 2018). The available copper concentration in our samples (0.46 – 42.17 mg Kg^–1^ with a median value of 2.89 mg Kg^–1^) was likely too low to be toxic to bacteria (Ippolito et al., 2011; Nunes et al., 2016); the potentiality of methanobactin to participate in signal transduction and copper transport (DiSpirito et al., 2016) and the significant relationships between ACu and topological features indicated that copper ions at low level may act as a limiting element mediating the links among bacteria taxa (Supplementary Figure 11). The influence of TN on fungal co-occurrence patterns may also reflect the critical role of TN in the nutrient availability of fungal taxa in the rhizosphere of *P. notoginseng* as mentioned above (Shi et al., 2020). In addition, we observed the more important role of geographic distances in explaining the local co-occurrence patterns of fungi and the deeper distance-decay relationship of fungal topological properties compared with bacteria (Figure 5C and Supplementary Figure 12), which further emphasized the importance of dispersal limitation in shaping co-occurrence structures.

## Conclusion

Based on a large-scale survey on rhizosphere microbiota across the cultivatable area of perennial medical plant *P. notoginseng* and comparative analyses, we systematically analyzed the macroecological patterns and community characters of two vital but different microorganisms in the rhizosphere. The assembly of fungal community was more driven by dispersal limitation relative to selection compared with the assembly of bacterial community; pH and TN mediated the balance between deterministic and stochastic assembly of bacteria and fungi, respectively. In addition, fungal communities exhibited potentially broader environmental thresholds and more modular co-occurrence patterns compared with bacteria. Our study emphasizes the importance of dispersal limitation in structuring rhizosphere microbiota and shaping the community features of ecologically distinct microorganisms. This knowledge can promote our understanding on the formation of plant-rhizosphere microbes holobionts and provides insights into a better utilization of these microbial communities.

## Data Availability Statement

The datasets presented in this study can be found in online repositories. The names of the repository/repositories and accession number(s) can be found below: https://www.ncbi.nlm.nih.gov/, PRJNA559079.

## Author Contributions

GZ did the formal analysis, software, visualization, writing – original draft, and writing – review and editing. GW did the investigation and writing – review and editing. FW and YW did the investigation and resources. ZC and MH did the resources. SJ did the methodology and writing – review and editing. LD did the conceptualization, writing - review and editing, and funding acquisition. SC did the project administration, funding acquisition, supervision, and writing - review and editing. All authors contributed to the article and approved the submitted version.

## Conflict of Interest

FW is employed by Wenshan Miaoxiang Notoginseng Technology, Co., Ltd. The remaining authors declare that the research was conducted in the absence of any commercial or financial relationships that could be construed as a potential conflict of interest.

## Publisher’s Note

All claims expressed in this article are solely those of the authors and do not necessarily represent those of their affiliated organizations, or those of the publisher, the editors and the reviewers. Any product that may be evaluated in this article, or claim that may be made by its manufacturer, is not guaranteed or endorsed by the publisher.

## Footnotes

1
www.worldclim.org

2
https://github.com/JTFouquier/ghost-tree-trees
