# Supplementary material for: Dispersal Limitation Plays Stronger Role in the Community Assembly of Fungi Relative to Bacteria in Rhizosphere Across the Arable Area of Medicinal Plant
Source: Front Microbiol. 2021 Aug 16;12:713523. doi: 10.3389/fmicb.2021.713523 (PMC8415459; doi:10.3389/fmicb.2021.713523)

**Dispersal limitation plays stronger role in the community assembly of fungi relative to bacteria in rhizosphere across the arable area of medicinal plant**

Guozhuang Zhang^a,1^, Guangfei Wei^a,1^, Fugang Wei^b^, Zhongjian Chen^c^, Mingjun He^d^, Shuo Jiao^e^, Yong Wang^c^, Linlin Dong^a,^ ^⁎^, Shilin Chen^a, ⁎⁎^

^a^ *Key Laboratory of Beijing for Identification and Safety Evaluation of Chinese Medicine, Institute of Chinese Materia Medica, China Academy of Chinese Medical Sciences, Beijing 100700, China*

^b^ *Wenshan Miaoxiang Notoginseng Technology, Co., Ltd., Wenshan 663000, China*

^c^ *Institute of Sanqi Research, Wenshan University, Wenshan 663000, China*

^d^ *Hainan Branch Institute of Medicinal Plant, Chinese Academy of Medical Sciences & Peking Union Medical College, Wanning 571533, China*

^e^ *State Key Laboratory of Crop Stress Biology in Arid Areas, College of Life Sciences, Northwest A & F University, Yangling 712100, China*

^⁎^ **Corresponding author**

TEL: (+86) 18911917789; fax: (+86) 1062899776; email: [lldong@icmm.ac.cn](mailto:lldong@icmm.ac.cn)

^⁎⁎^ **Corresponding author**

TEL: (+86) 1057203877; fax: (+86) 1062899776; email: slchen@icmm.ac.cn

**Author contributions**

^1^These authors contributed equally.

**Table S1.** Reserved variables, explained variations and *P*-values of each part in variation partition analysis (VPA).

| **Model** | **Part** | **Explained variations & *P*-values** | | **Variables** |
| --- | --- | --- | --- | --- |
|  |  | Total | Pure |  |
| **Bacteria** | Soil | 0.45 *P* =0.001 | 0.19 *P* =0.001 | pH; TN; EMg; AP; AFe; TP; OM; AN; AS; AK; TK; AMn; ECa; SC; Cl; ACu; AB; AZn |
|  | Climate | 0.18 *P* =0.001 | 0.03 *P* =0.001 | MAT; MAP; Ele |
|  | Space | 0.30 *P* =0.001 | 0.08 *P* =0.001 | PCNM1; PCNM2; PCNM3; PCNM4; PCNM5; PCNM8; PCNM9 |
|  | Residuals | 0.41 | | - |
| **Fungi** | Soil | 0.38 *P* =0.001 | 0.20 *P* =0.001 | pH; TN; EMg; AP; AFe; TP; OM; AN; AS; AK; TK; AMn; ECa; SC; Cl; ACu; AB; AZn |
|  | Climate | 0.14 *P* =0.001 | 0.03 *P* =0.001 | MAT; MAP; Ele |
|  | Space | 0.27 *P* =0.001 | 0.11 *P* =0.001 | PCNM1; PCNM2; PCNM3; PCNM4; PCNM5; PCNM7; PCNM8; PCNM9; PCNM11 |
|  | Residuals | 0.45 | | - |

The abbreviation of environmental factors was defined in ***Materials and Methods***.

**Table S2.** Fitness results for all variables in non-metric multi-dimensional scaling (NMDS) ordination

| **Environmental and**  **biotic variables** | | **Bacteria** | | **Fungi** | | **Note** |
| --- | --- | --- | --- | --- | --- | --- |
|  |  | **R^2^** | ***P*-value** | **R^2^** | ***P*-value** |  |
| **Edaphic**  **factors** | **pH** | **0.7547** | **0.001** | **0.2576** | **0.001** |  |
|  | **TN** | **0.4130** | **0.001** | **0.2409** | **0.001** |  |
|  | **AP** | **0.2280** | **0.001** | **0.2403** | **0.001** |  |
|  | **TP** | **0.1064** | **0.013** | 0.0512 | 0.137 |  |
|  | **AK** | **0.2018** | **0.001** | **0.0970** | **0.027** |  |
|  | **TK** | 0.0301 | 0.323 | 0.0216 | 0.465 |  |
|  | **AB** | **0.1669** | **0.001** | 0.0385 | 0.2081 |  |
|  | **AMn** | **0.1631** | **0.002** | **0.3164** | **0.001** |  |
|  | **AS** | **0.1473** | **0.004** | 0.0746 | 0.052 |  |
|  | **AFe** | **0.4872** | **0.001** | **0.3767** | **0.001** |  |
|  | **ACu** | **0.0966** | **0.024** | 0.0337 | 0.286 |  |
|  | **AZn** | 0.0628 | 0.09 | **0.1565** | **0.003** |  |
|  | **Cl** | 0.0294 | 0.337 | **0.1336** | **0.007** |  |
|  | **SC** | 0.0002 | 0.995 | 0.0091 | 0.718 |  |
|  | **OM** | **0.3966** | **0.001** | **0.3759** | **0.001** | - |
|  | **AN** | **0.2722** | **0.001** | **0.3230** | **0.001** | - |
|  | **ECa** | **0.4503** | **0.001** | **0.0674** | **0.073** | - |
|  | **EMg** | **0.4132** | **0.001** | 0.0281 | 0.342 | - |
| **Climatic**  **factors** | **MAP** | 0.0642 | 0.101 | 0.0170 | 0.513 |  |
|  | **MAT** | 0.0545 | 0.144 | **0.1237** | **0.005** |  |
|  | **Ele** | 0.0164 | 0.554 | **0.2818** | **0.001** | - |
| **Spatial**  **factors** | **Longitude** | **0.2316** | **0.001** | **0.3930** | **0.001** |  |
|  | **Latitude** | **0.1979** | **0.001** | 0.0025 | 0.917 |  |

**Edaphic variables with R^2^ > 0.2 were displayed in Fig. 1. ' - ' represents variables were ignored due to strong collinearity.** The abbreviation of environmental factors was defined in ***Materials and Methods***.

**Table S3.** Mantel test between phylogenetic turnover (*β*NTI) and distance matrices of environmental variables as well as geographic distance

| **Distance matrices of variables** | | **Mantel *r***  **Bacteria *β*NTI** | **Mantel *r***  **Fungal *β*NTI** |
| --- | --- | --- | --- |
| Group | Variable |  |  |
| Space | Geographic distance | **-0.27860***** | 0.02098 |
| Climate | MAT | -0.09457 | 0.01042 |
|  | MAP | **-0.07297*** | 0.00740 |
|  | Ele | -0.07032 | 0.06864 |
| Soil | pH | **-0.17587***** | 0.04352 |
|  | OM | **-0.32519***** | -0.01624 |
|  | TN | **-0.26420***** | **-0.10833**** |
|  | TP | 0.08602 | -0.05000 |
|  | TK | -0.04445 | -0.02904 |
|  | AN | **-0.26297***** | -0.02633 |
|  | AP | 0.10522 | 0.01288 |
|  | AK | **0.17957**** | 0.07027 |
|  | Cl | 0**.14021*** | 0.05249 |
|  | AB | 0.072525 | -0.03069 |
|  | AS | -0.03437 | -0.02364 |
|  | ECa | 0.11168 | -0.04810 |
|  | EMg | 0.01607 | -0.01967 |
|  | ACu | 0.10914 | **-0.11558*** |
|  | AZn | 0.03963 | 0.05060 |
|  | AFe | **-0.35765***** | 0.01988 |
|  | AMn | 0.10321 | **0.12828*** |
|  | SC | -0.0572 | -0.05435 |

* *P* < 0.05; ** *P* < 0.01; *** *P* < 0.001. The abbreviation of environmental factors was defined in ***Materials and Methods***.

**Table S4.** Partial mantel test results showing comparison between *β*NTI and one independent distance matrices, while controlling for all other explanatory distance matrices constant.

| **Taxonomy** | **Distance matrices of**  **variables** | **Controlling for** | **Partial Mantel *r*** |
| --- | --- | --- | --- |
| Bacteria | Geographic distance | All environmental factors | 0.00571 |
|  | pH | Geographic distance + Other environmental factors | **-0.22601***** |
|  | OM | Geographic distance + Other environmental factors | **-0.14960**** |
|  | TN | Geographic distance + Other environmental factors | -0.00163 |
|  | AN | Geographic distance + Other environmental factors | 0.078171 |
|  | AK | Geographic distance + Other environmental factors | **0.06953*** |
|  | AFe | Geographic distance + Other environmental factors | -0.07000 |
|  | Cl | Geographic distance + Other environmental factors | **0.1158**** |
|  | MAP | Geographic distance + Other environmental factors | -0.04067 |
| Fungi | Geographic distance | All environmental factors | -0.04247 |
|  | TN | Geographic distance + Other environmental factors | **-0.18025***** |
|  | ACu | Geographic distance + Other environmental factors | -0.04504 |
|  | AMn | Geographic distance + Other environmental factors | 0.02481 |

* *P* < 0.05; ** *P* < 0.01; *** *P* < 0.001. The abbreviation of environmental factors was defined in ***Materials and Methods***.

**Fig. S1** Species accumulation curves of bacteria and fungi at local (A) and global (B) level. The x axis represents the number of sequences. The y axis represents the number of OTUs.


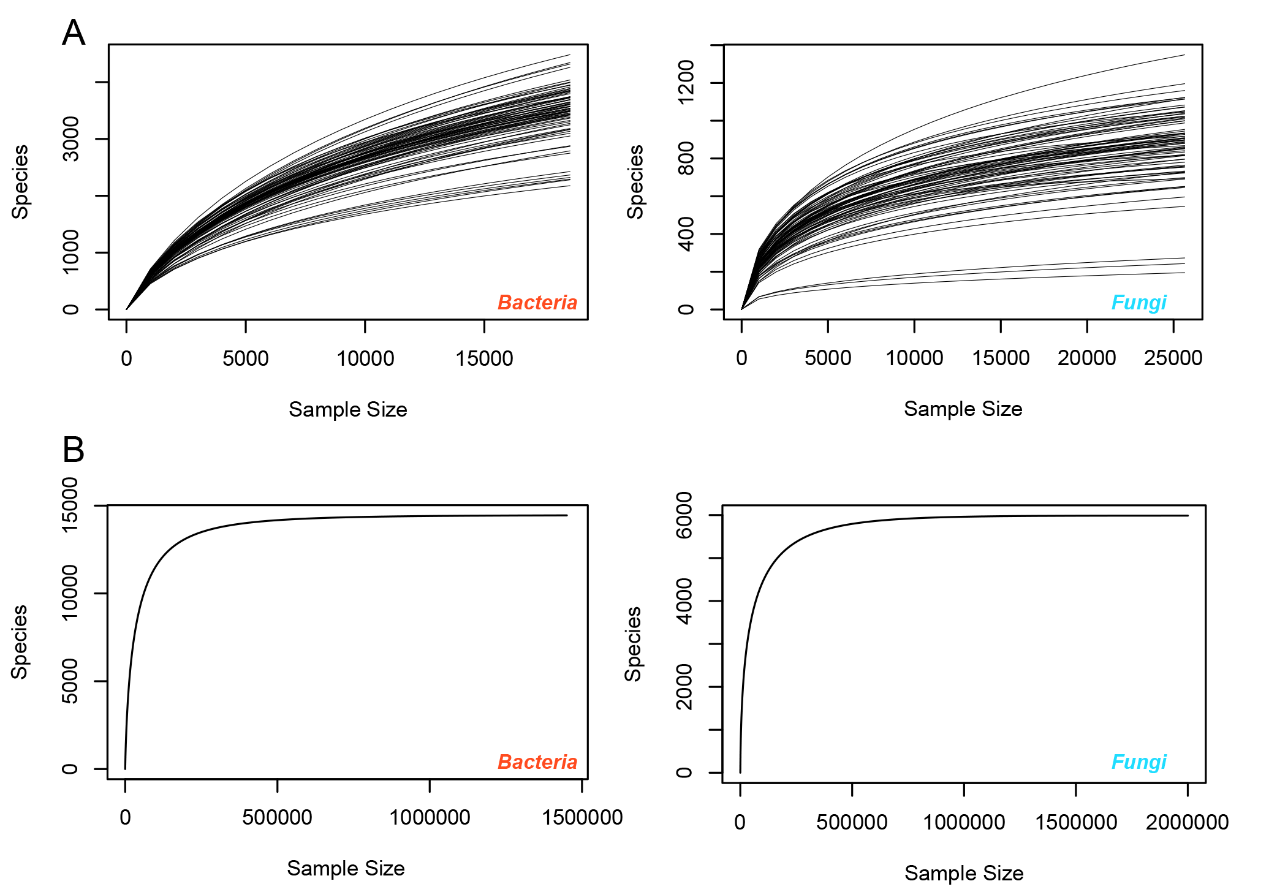


**Fig. S2** Variable clustering plot showing the collinearity among measured variables. Red line represents the selection threshold. The abbreviation of environmental factors was defined in ***Materials and Methods***.


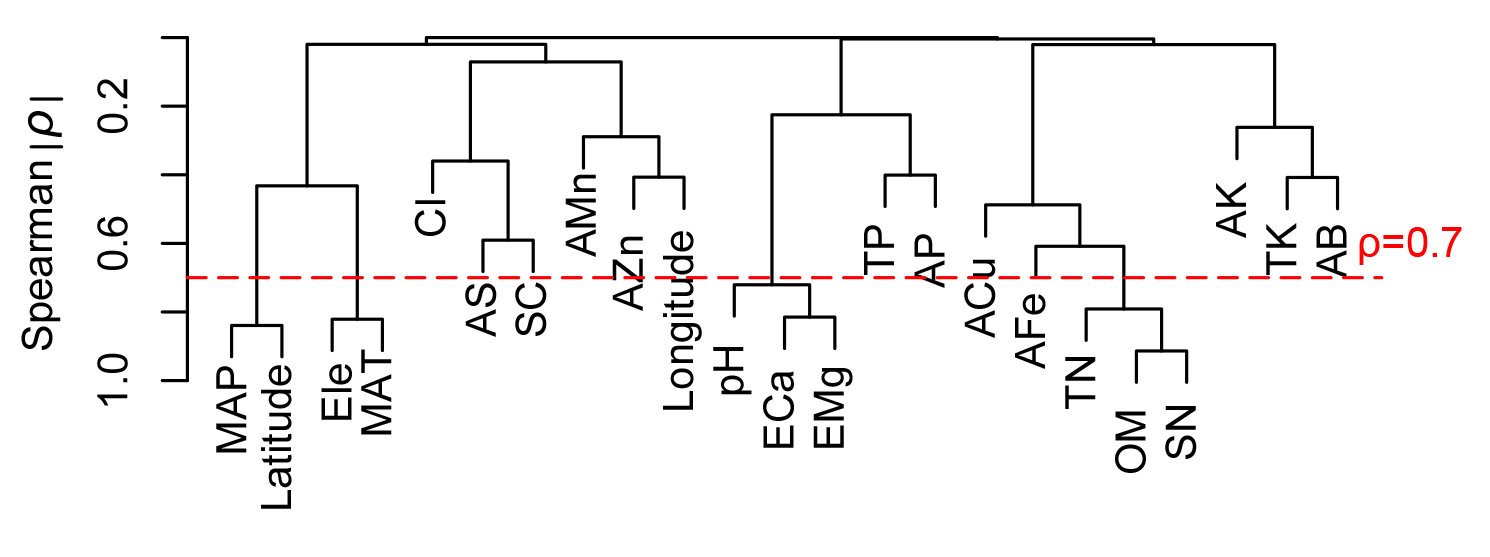


**Fig. S3** Phylogenetic Mantel correlograms illustrating the phylogeny signals of bacteria and fungi. Solid and open squares represent significant and non-significant correlations, respectively, relating between-OTU niche preferences for pH to between-OTU phylogenetic distances.


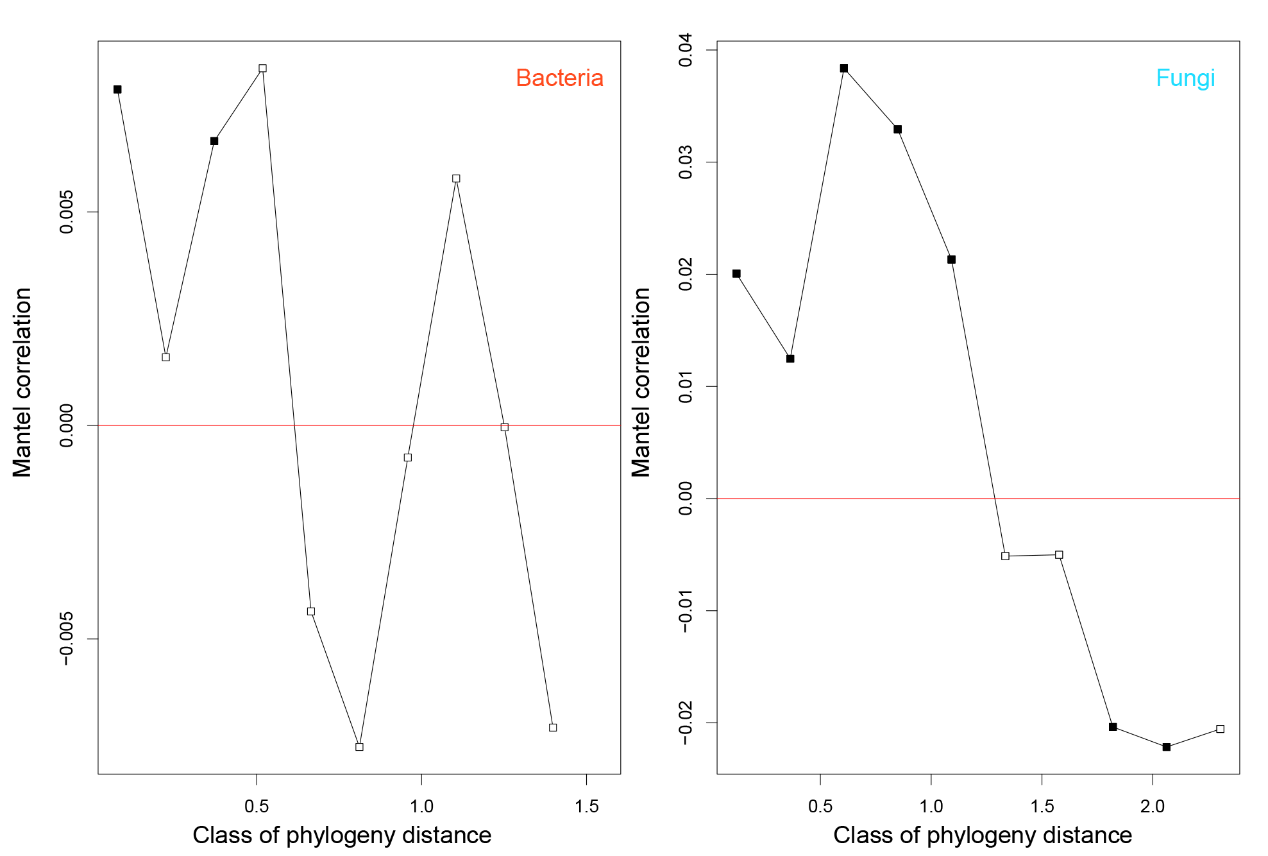


**Fig. S4** The abundance ratio of OTUs which responded to key variables in each phylum and functional guilds of bacteria or fungi. Symbol size represents the proportion of cumulated abundance of OTUs which showed negative or positive response to one variable in the total abundance of corresponding taxonomic or functional groups. (**A**) Bacteria phylum. (**B**) Fungi phylum. (**C**) Fungi functional guilds.


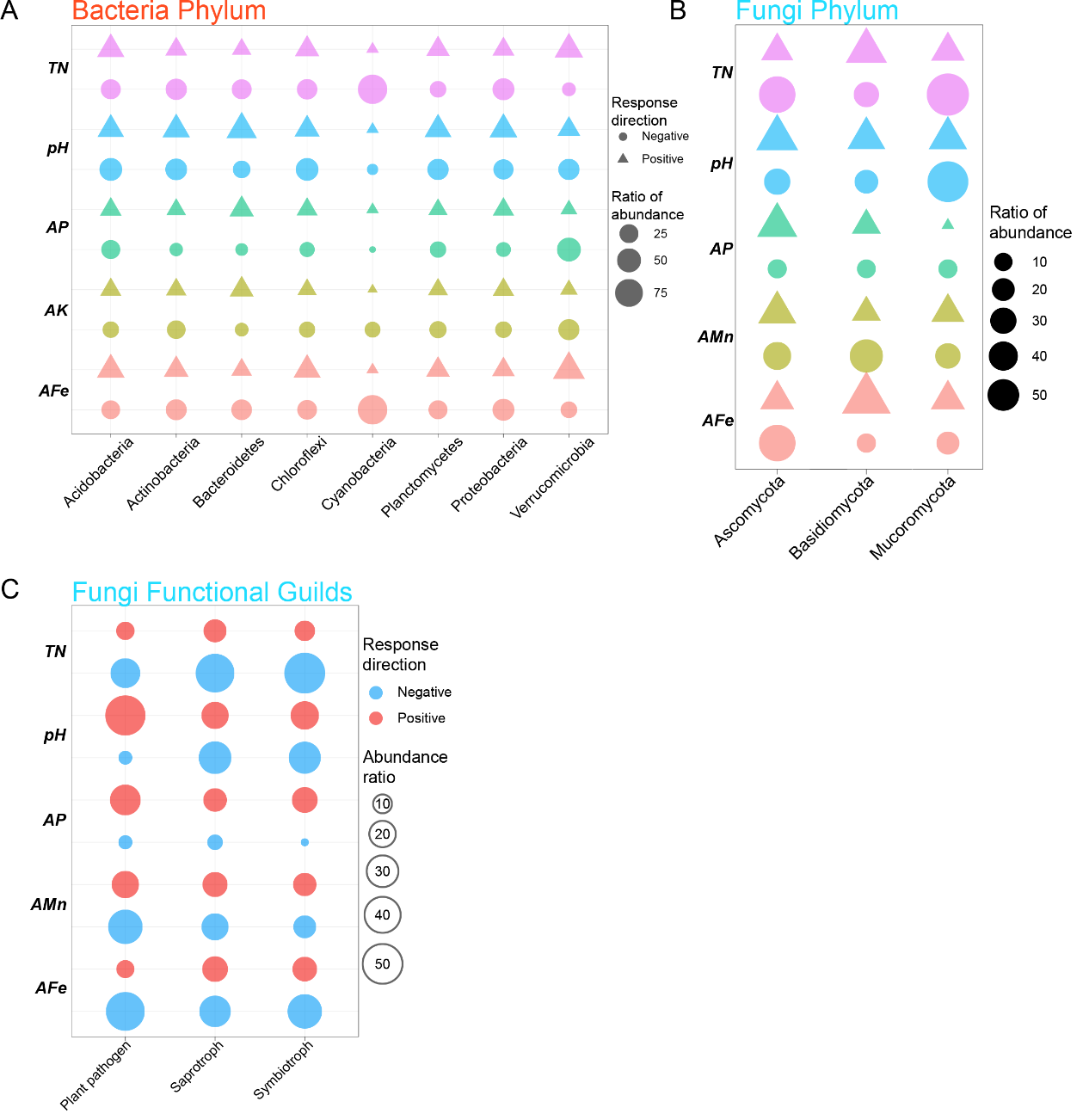


**Fig. S5** Contributions of measured variables to the abundance distribution patterns of dominant phyla and fungal functional guilds. Circle size represents the variable importance identified by multiple regression modeling and variance decomposition analysis. Tile colors represent Spearman correlations. The abbreviations of variables accord to the ***Materials and Methods***. Bar plots on the right represent the variance explained by corresponding models.

**
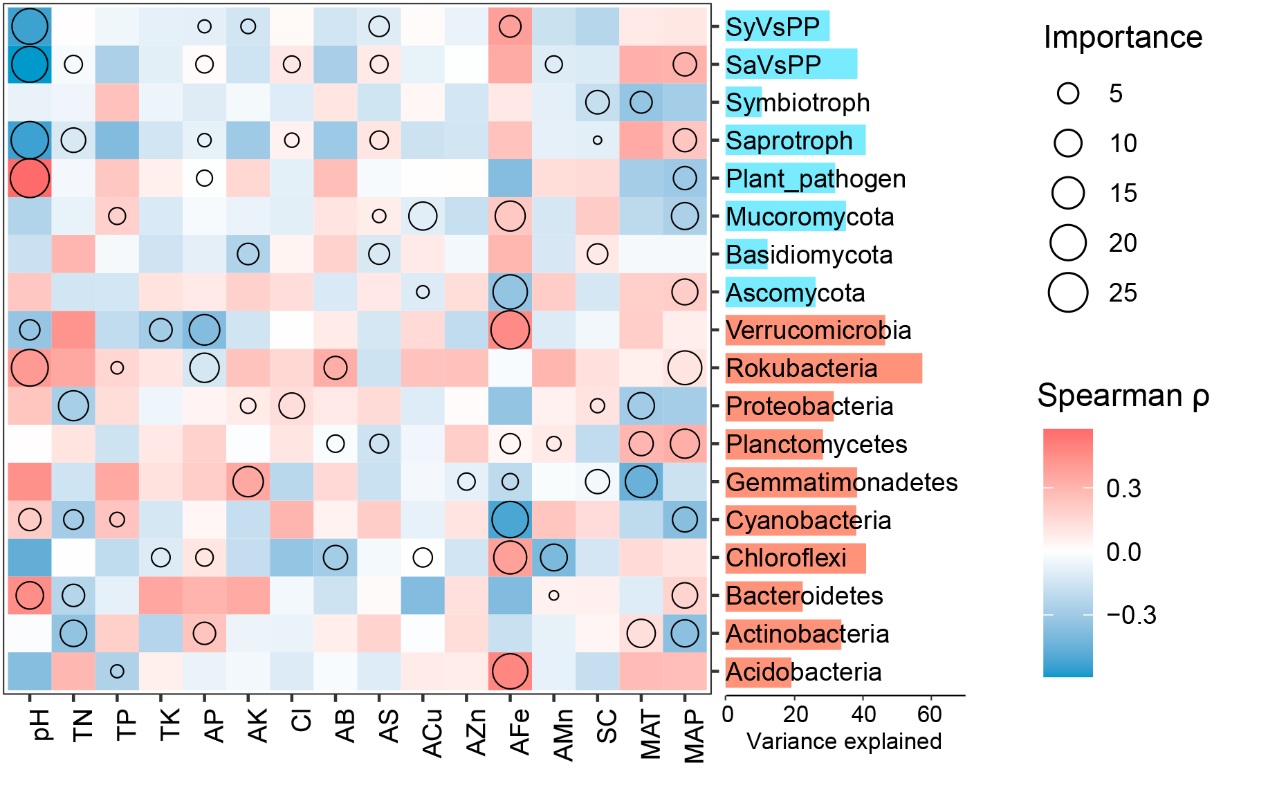
**

**Fig. S6** Ecological processes underlying community assembly estimated by Stegen’s null model.

The relative contributions of different ecological processes to the community assembly of (A) bacteria, (B) fungi based on ghost_tree, (C) fungi based on taxonomy_to_tree. (D) The comparison of SDER between fungi and bacteria using Stegen’s framework, in which the *β*NTI of fungi was calculated based on the phylogeny constructed through taxonomy_to_tree.pl


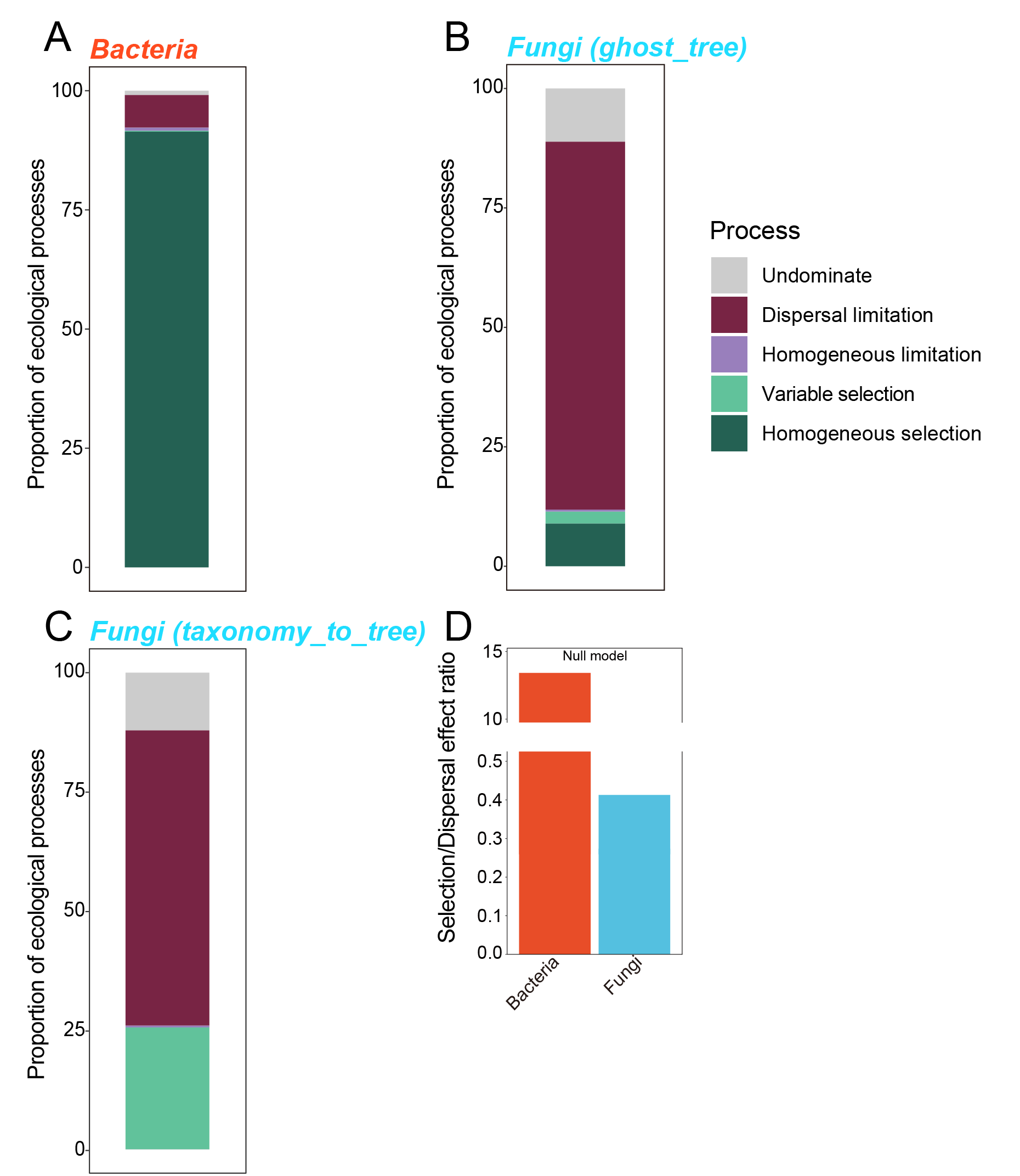


**Fig. S7** The relative importance of ecological processes identified through RC_Bray_. |RC_Bray_| < 0.95 represents the contribution of stochastic process, |RC_Bray_| > 0.95 indicates a deterministic assembly.

**
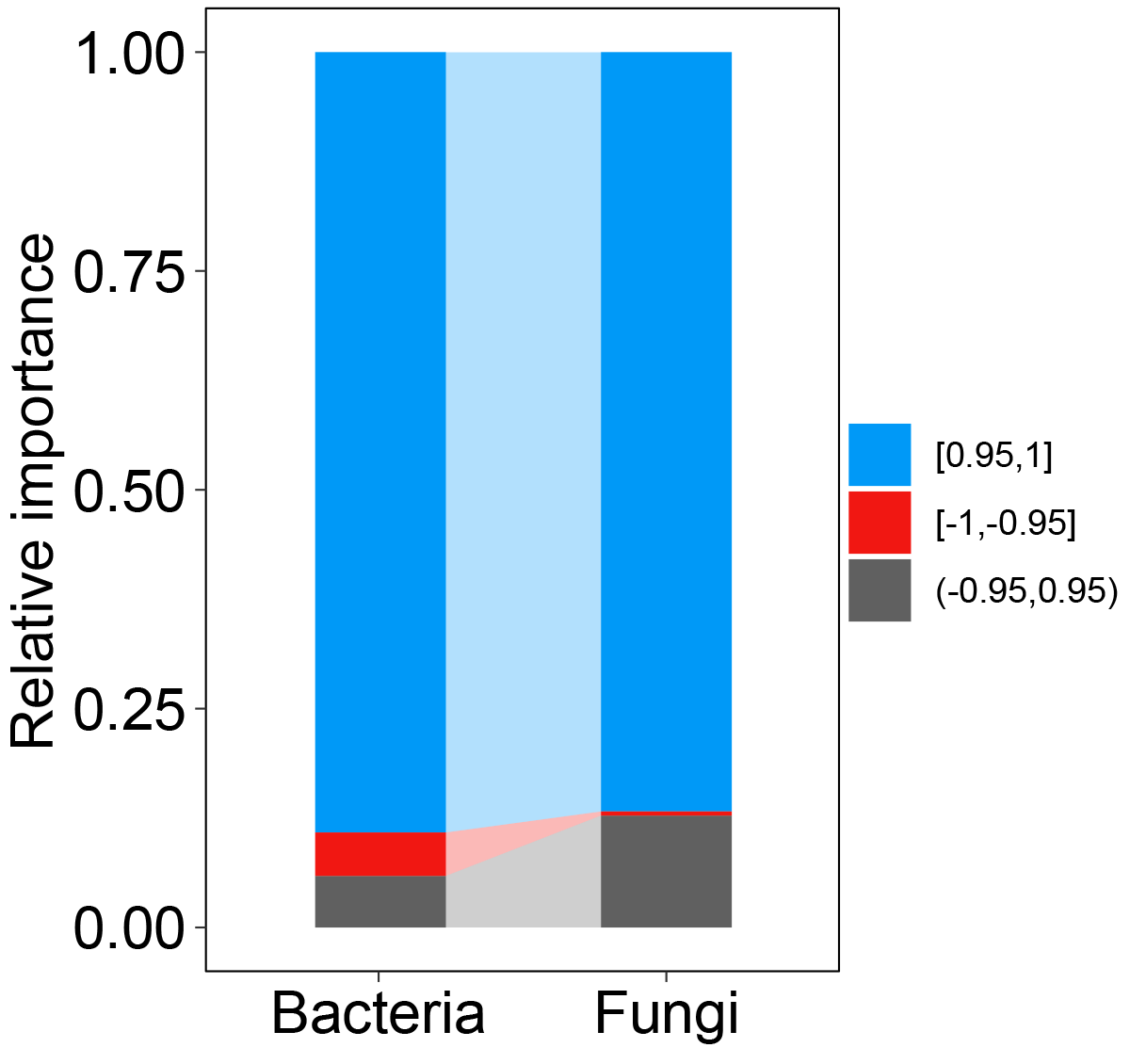
**

**Fig. S8** The features of metacommunity co-occurrence networks. (**A**) The degree distribution of cross-domain, bacterial, and fungal networks. Red line represents the maximum-likelihood fitting of exponential distribution. *P* values were obtained through Kolmogorov-Smirnov test. (**B**) Dot plots illustrating the relationships between the relative abundance and degree of OTUs. Lines represent the ordinary least-square regression. (**C**) The comparisons of node level topological properties between bacterial and fungal nodes. The significance of difference is estimated by Wilcoxon rank-sum test. ***, *P* < 0.001.


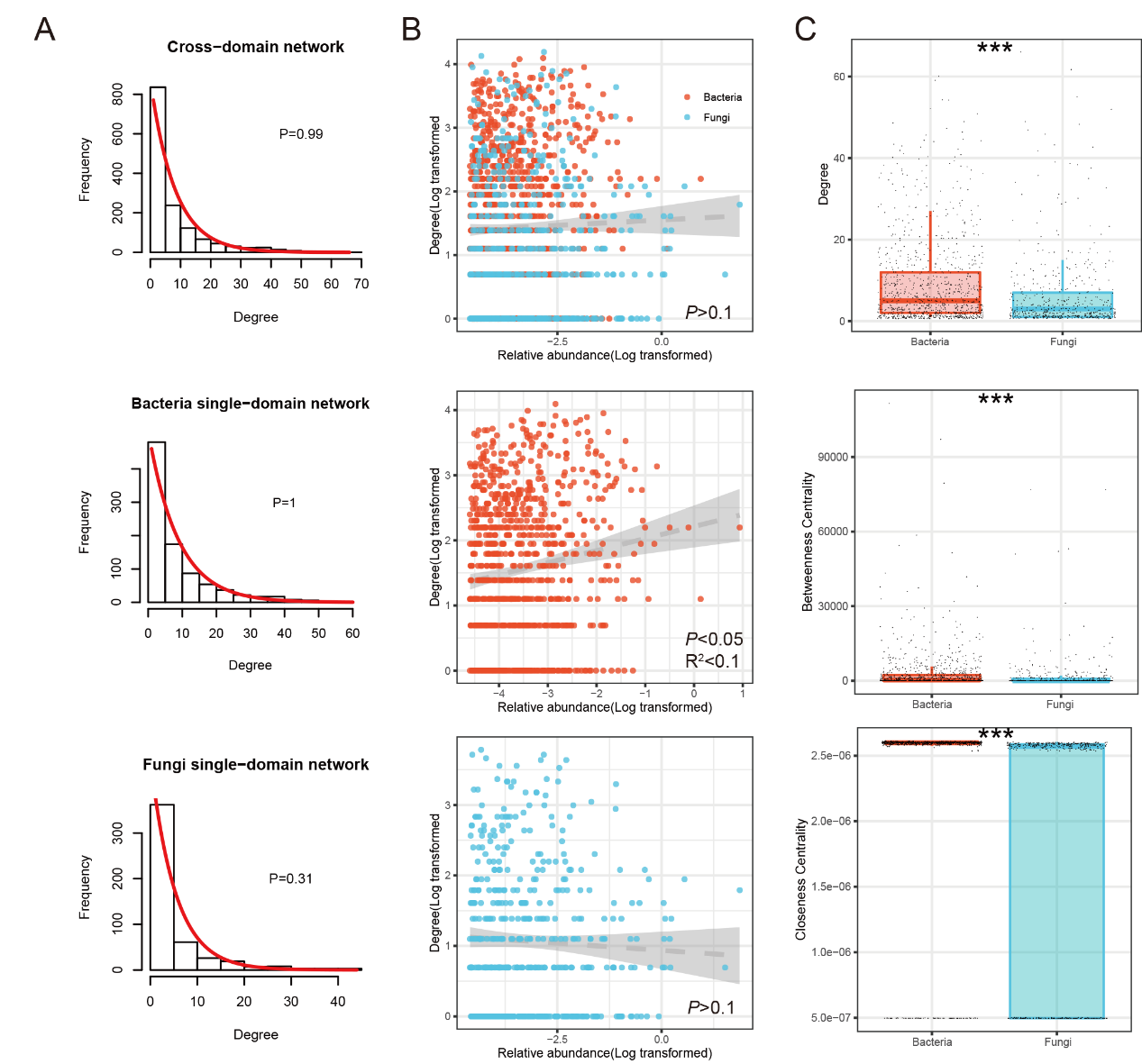


**Fig. S9** The composition and bacteria-fungi links in cross-domain metacommunity network. (**A**) The composition of main modules. Nodes are colored according to corresponding modules. Loop graphs on the right represent the composition of different modules at class level. (**B**) The links between bacterial and fungal nodes. Colors are mapped to bacterial phyla and fungal class, respectively.


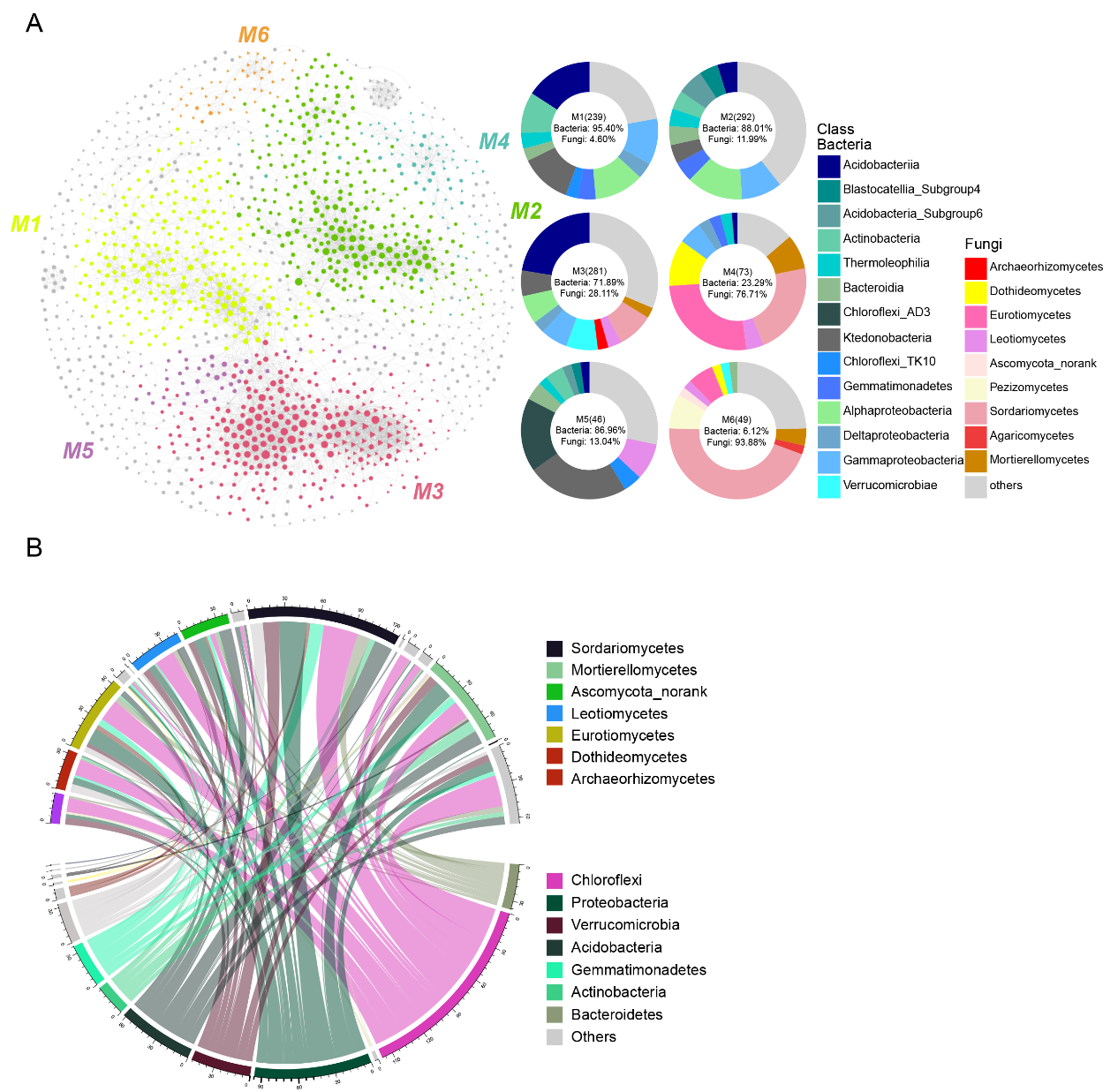


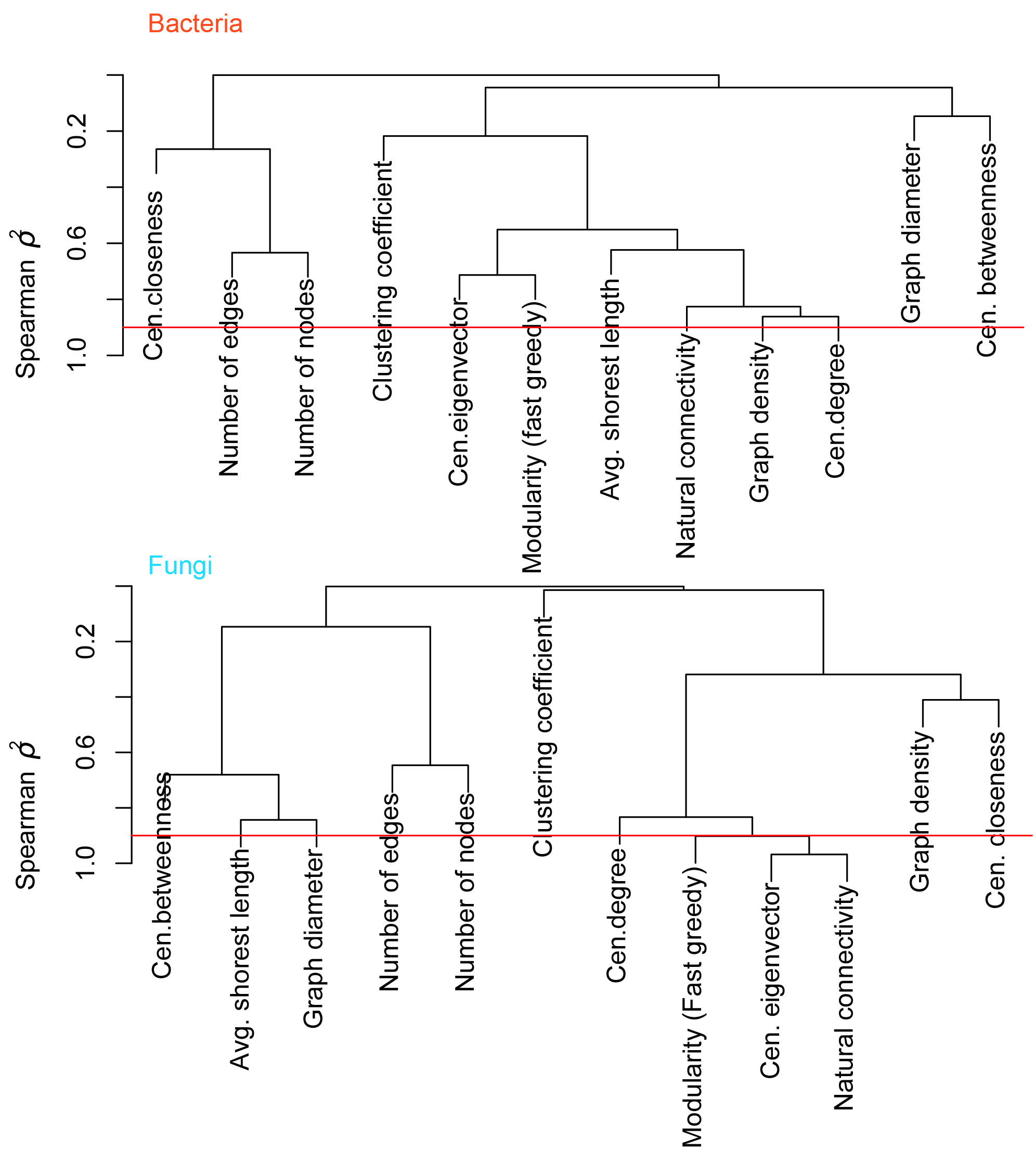
**Fig. S10** The clustering plot of local network-level topological features of bacteria and fungi. Red lines represent the threshold. Avg.: average; Cen.: centrality.

**Fig. S11** Spearman correlations among local network features and measured variables. The abbreviations of variables accord to the *Materials and Methods*. Avg.: average; Cen.: centrality. *, *P* < 0.05; **, *P* < 0.01; ***, *P* < 0.001.


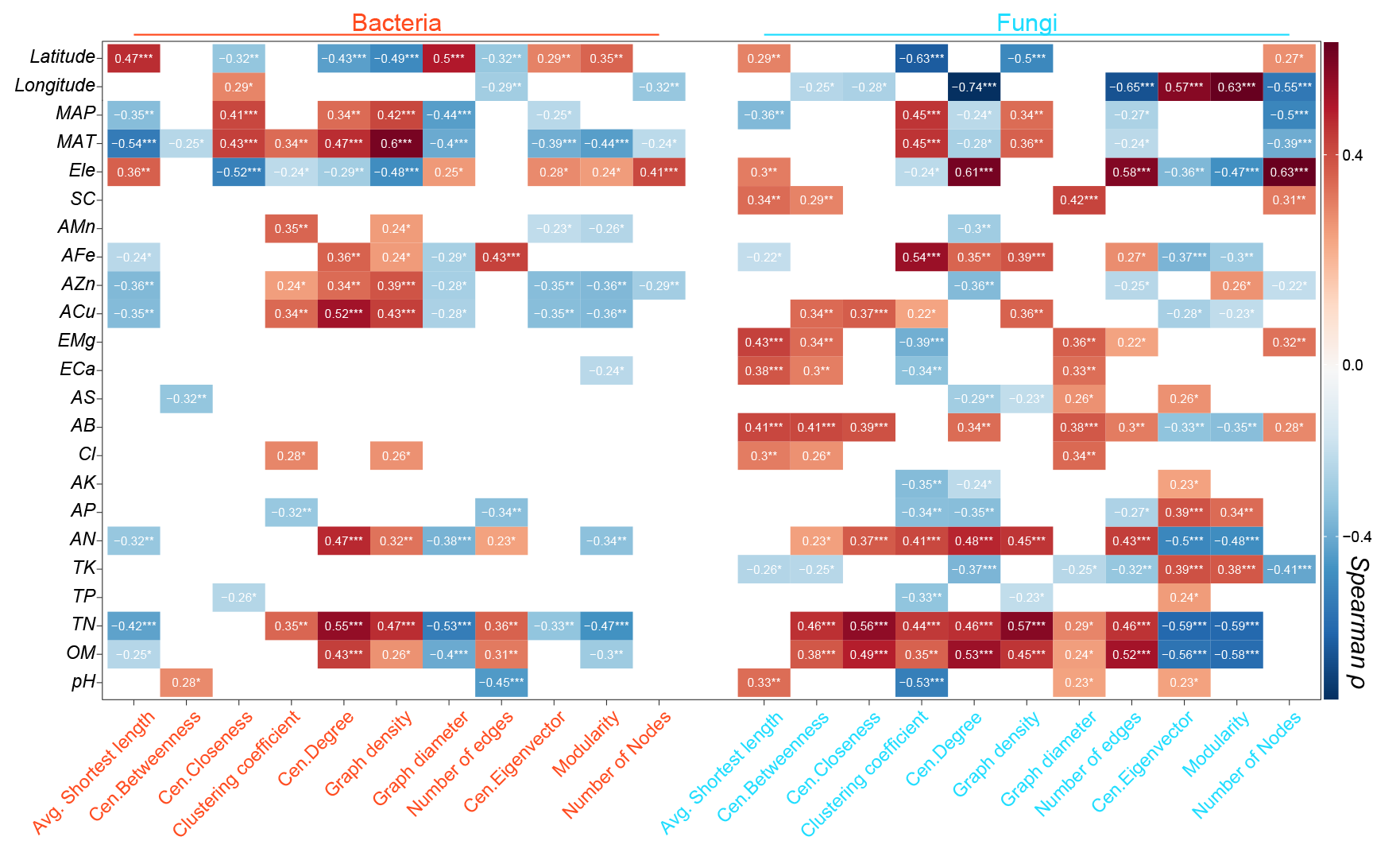


**Fig. S12** The distance-decay relationships of topological features of local networks against geographic distance. Vertical axis represents the Euclidean similarity standardized by following formula: 1 / (1-distance). Solid lines represent the ordinary least-square regression.


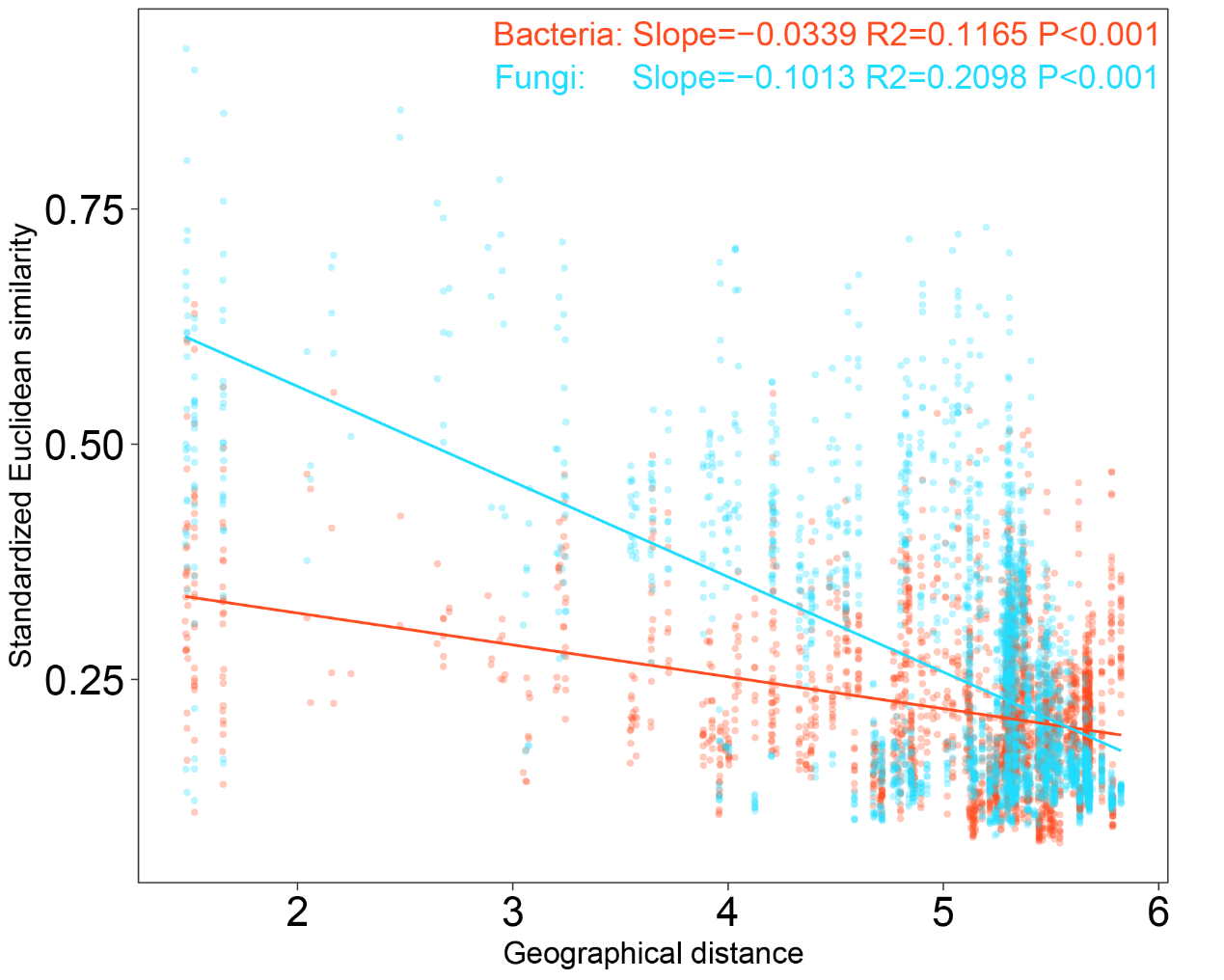

Supplement: Supplementary file 1 [file Table_1.DOCX]
